# Comparative Plasma Lipidome between Human and Cynomolgus Monkey: Are Plasma Polar Lipids Good Biomarkers for Diabetic Monkeys?

**DOI:** 10.1371/journal.pone.0019731

**Published:** 2011-05-04

**Authors:** Guanghou Shui, Jeffrey William Stebbins, Buu Duyen Lam, Wei Fun Cheong, Sin Man Lam, Francine Gregoire, Jun Kusonoki, Markus R. Wenk

**Affiliations:** 1 Department of Biochemistry, Yong Loo Lin School of Medicine, National University of Singapore, Singapore, Singapore; 2 Life Sciences Institute, National University of Singapore, Singapore, Singapore; 3 Department of Biological Sciences, Yong Loo Lin School of Medicine, National University of Singapore, Singapore, Singapore; 4 Lilly Singapore Center for Drug Discovery Pte Ltd., Singapore, Singapore; Governmental Technical Research Centre of Finland, Finland

## Abstract

**Background:**

Non-human primates (NHP) are now being considered as models for investigating human metabolic diseases including diabetes. Analyses of cholesterol and triglycerides in plasma derived from NHPs can easily be achieved using methods employed in humans. Information pertaining to other lipid species in monkey plasma, however, is lacking and requires comprehensive experimental analysis.

**Methodologies/Principal Findings:**

We examined the plasma lipidome from 16 cynomolgus monkey, *Macaca fascicularis*, using liquid chromatography coupled with mass spectrometry (LC/MS). We established novel analytical approaches, which are based on a simple gradient elution, to quantify polar lipids in plasma including (i) glycerophospholipids (phosphatidylcholine, PC; phosphatidylethanolamine, PE; phosphatidylinositol, PI; phosphatidylglycerol, PG; phosphatidylserine, PS; phosphatidic acid, PA); (ii) sphingolipids (sphingomyelin, SM; ceramide, Cer; Glucocyl-ceramide, GluCer; ganglioside mannoside 3, GM3). Lipidomic analysis had revealed that the plasma of human and cynomolgus monkey were of similar compositions, with PC, SM, PE, LPC and PI constituting the major polar lipid species present. Human plasma contained significantly higher levels of plasmalogen PE species (p<0.005) and plasmalogen PC species (p<0.0005), while cynomolgus monkey had higher levels of polyunsaturated fatty acyls (PUFA) in PC, PE, PS and PI. Notably, cynomolgus monkey had significantly lower levels of glycosphingolipids, including GluCer (p<0.0005) and GM_3_ (p<0.0005), but higher level of Cer (p<0.0005) in plasma than human. We next investigated the biochemical alterations in blood lipids of 8 naturally occurring diabetic cynomolgus monkeys when compared with 8 healthy controls.

**Conclusions:**

For the first time, we demonstrated that the plasma of human and cynomolgus monkey were of similar compositions, but contained different mol distribution of individual molecular species. Diabetic monkeys exhibited decreased levels of sphingolipids, which are microdomain-associated lipids and are thought to be associated with insulin sensitivity. Significant increases in PG species, which are precursors for cardiolipin biosynthesis in mitochondria, were found in fasted diabetic monkeys (n = 8).

## Introduction

Diabetes is a worldwide public health challenge. WHO estimates that more than 220 million people worldwide have diabetes and 1.1 million people died from the disease in 2005. Its incidence is increasing rapidly due to aging, urbanization, and increasing prevalence of obesity and sedentary lifestyle [1]. Furthermore, it is estimated that over 30% of diabetic population remain undiagnosed [2]. On another note, despite the availability of many anti-diabetic agents, current treatment goals are often unmet and are often constrained by weight gain, tolerability, safety and gastrointestinal intolerance The various limitations aforementioned demand an immediate and ideal therapeutic intervention for the disease. The development of a reliable diagnostic test would therefore not only be highly beneficial for clinical purposes, but also for the testing of response to new drug in animal models.

Obesity is the single most important contributor to insulin resistance [3]. Elevated levels of plasma lipids had been shown to cause changes in cellular lipid metabolism that would lead to intracellular lipid accumulation, compromising other organ functions as a result. Increasing emphasis has been placed on the measurement of triglycerides, non-esterified fatty acids (NEFAs) and cholesterol levels in the plasma as well as in specific tissues susceptible to lipid accumulation that would affect normal physiologic function as a result [4]. Recently, inhibition of sphingolipid synthesis was shown to increase insulin sensitivity in cultured cells and rodent models [5], [6], [7], [8], [9], [10], [11]. Mice lacking the GM_3_ synthase (GMS) have elevated insulin sensitivity [12]. Moreover, patients with Type I Gaucher disease, a lysosomal storage disease caused by a failure to degrade glucosylated ceramides, are insulin-resistant [13]. The “membrane microdomain ortho-signalling therapy” represents an emerging concept that opens a new therapeutic window for the treatment of metabolic disorders. It proposes that metabolic disorders can arise as a result of disorganizations in membrane microdomains due to the aberrant expression of gangliosides [14]. These findings cumulatively suggest that quantitative changes in sphingolipids can be potentially relevant modulators of insulin action.

Non-human primates (NHPs) are more closely related to human compared to rodents. As such, they are currently being considered as the primary model for evaluation of diabetic drugs [15], [16], [17]. Nevertheless, information on the lipid profiles of monkey plasma has not been previously reported, probably due to a lack in the state-of-the-art techniques for lipid analysis and the lower accessibility of monkeys to academic research. A quantitative analysis of monkey plasma lipids could therefore provide important biological insights into the understanding of metabolic syndrome. Technological advancements, most notably in liquid chromatography and mass spectrometry, allow sensitive and highly selective analysis of lipids with diverse chemical composition and in complex mixtures [18], [19]. In particular, the introduction of tandem MS has spurred the development of various quantitative approaches, such as precursor ion scanning, neutral loss scanning, and MRM scanning for the targeted lipidomic analysis of complex samples[20], [21], [22]. Therefore, the development of lipid biomarkers for diabetes using mass spectrometry would facilitate the diagnostic process and enable more effective treatment monitoring of the disease. To our knowledge, this is the first study carried out to characterize monkey plasma lipids in parallel with human plasma lipids. This study also aims to investigate the potential lipid biomarker(s) for diabetes using monkey as a scale-down model for potential applications in human drug evaluation.

## Results

**Figure 1:**
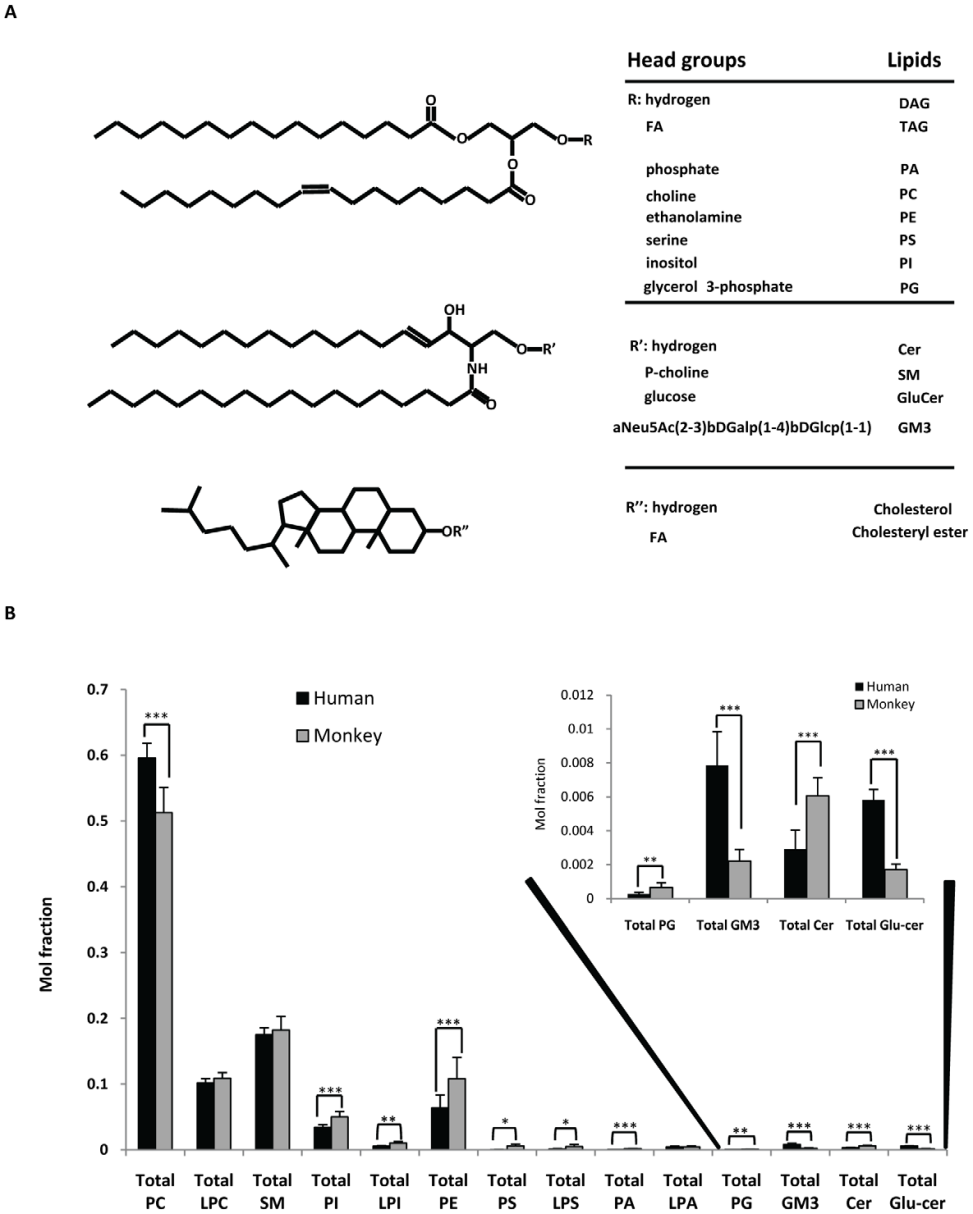
Major lipid species in human and monkey. (A) Chemical structure of plasma lipids. (B) Comparison of total mol fraction of phospholipid and sphingolipids species in Human (n = 10) and normal, fed Monkey plasma (n = 8). Insert, PG, GM3, Cer, Glucer. * p<0.05, ** p<0.005, *** p<0.0005.

Characterization of monkey plasma lipids using precursor ion scans (PreIS) indicated that the lipid species in monkey and human plasma are similar. This validated our approach in using similar analytical methods for the analyses of plasma samples from the two species. Here, we used a simple LC gradient elution to separate various classes of polar lipids (Figure 1A), in the plasma before introduction into mass spectrometer for quantification. In negative ESI mode, PG, PE, PI, PS, PA and GM_3_ species eluted out of column in the stated order with good separation. In positive ESI mode, Cer species flowed out of the column first, subsequently followed by GluCers, PCs and SMs. The various MRM transitions for all lipid species were monitored and their intensities measured [23]. The quantity of individual species was calculated by comparing its measured intensity with that of spiked internal standards. Primarily, we focused our analysis on phospholipids and sphingolipids, in particular for glycosphingolipids such as ganglioside GM3 and GluCer species. For the first time, we are able to quantify individual ganglioside GM3 species in both human and monkey using synthetic C17-ganglioside GM3 standard and specific MRM transitions.

### Comparison of plasma lipid profiles between monkey and human

Analyses of total lipid fraction of individual classes of lipids were shown in Figure 1B. Similar to the trend observed in human, PC was the most abundant phospholipid species in monkey plasma, followed by SM, LPC, PE and PI (Figure 1B). PC was significantly higher in the plasma of human than monkey, while other phospholipid species including PI and PE displayed the opposite trend (Figure 1B). Among sphingolipid species measured in human and monkey plasma, GM3, Cer and GluCer were found to be significantly different in quantities (Figure 1B). The levels of GM3 and GluCer in human plasma were nearly fourfold of that in monkey plasma; while the level of Cer in monkey plasma was approximately double of that in human plasma. However, the level of SM was not found to be significantly different between the two species (Figure 1B, insert).

**Figure 2:**
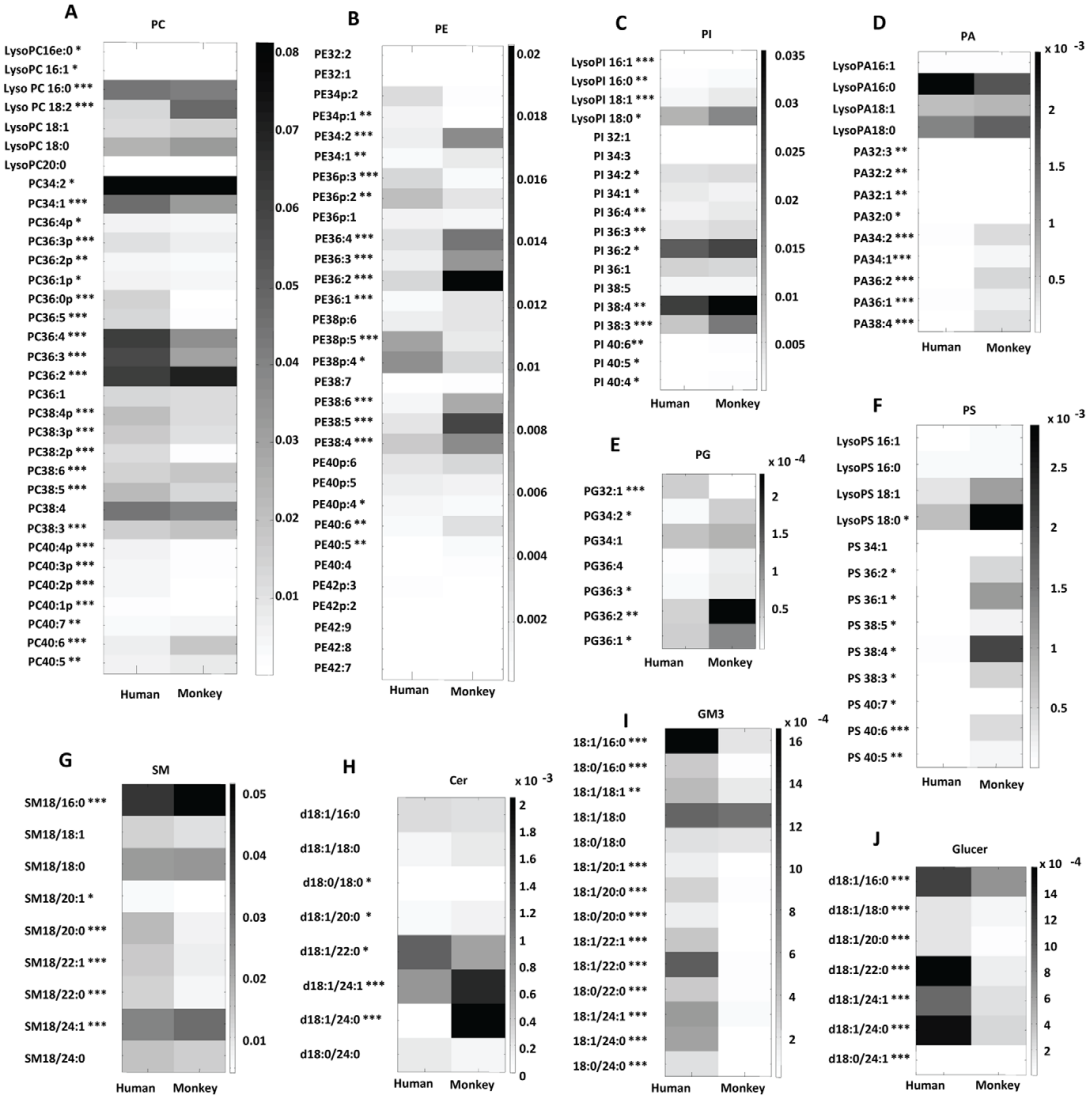
Heatplots of various polar lipids in normal human (n = 10) and normal, fed monkey plasma (n = 8) (A) PC, phosphatidylcholine; (B) PE, phosphatidylethanolamine; (C) PI, phosphatidylinositol; (D) PA, phosphatidic acid; (E) PG, phosphatidylglycerol; (F) PS, phosphatidylserine; (G) SM, sphingomyelin; (H), Cer, Ceramides; (I) GM3, ganglioside mannoside; (J), Glucer, glucosylceramides. * p<0.05, ** p<0.005, *** p<0.0005.

**Figure 3:**
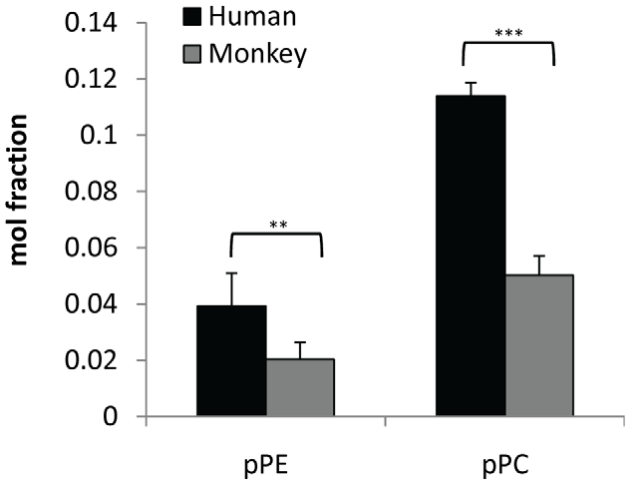
Comparison of mol fraction of plasmalogen lipids in both human (n = 10) and normal, fed Monkey plasma (n = 8). ** p<0.005, *** p<0.0005.

The levels of individual lipid species in human and monkey plasma were quantified and the differences were illustrated by the heat plots in Figure 2. Although human and monkey plasma contained the same lipid species, individual lipid species was found in different quantities. For instance, the levels of plasmalogen PE species (p<0.005) and plasmalogen PC species (p<0.0005) were significantly higher in human plasma than in monkey plasma (Figure 3), while the levels of polyunsaturated fatty acyls (PUFA), including PC40:6, PC40:5, PC38:6, PE40:6, PE40:5, PE38:6, PE38:5, PE38:4, were significantly higher in the plasma of monkey than that in human (Figure 2A, 2B). Ganglioside GM3, Cer and GluCer also showed dissimilar trend in the distribution of each lipid species (Figure 2H, 2I, 2J).

TAG profiles in human and monkey plasma demonstrated that monkey plasma contained higher levels of TAG 52:5, 52:4, 56:8, 56:7, 56:6, 58:10, 58:9, 58:8, 58:7 and 58:6 (Figure S1).

### Changes in plasma lipidome of diabetic monkeys under fed and fasting conditions

**Figure 4:**
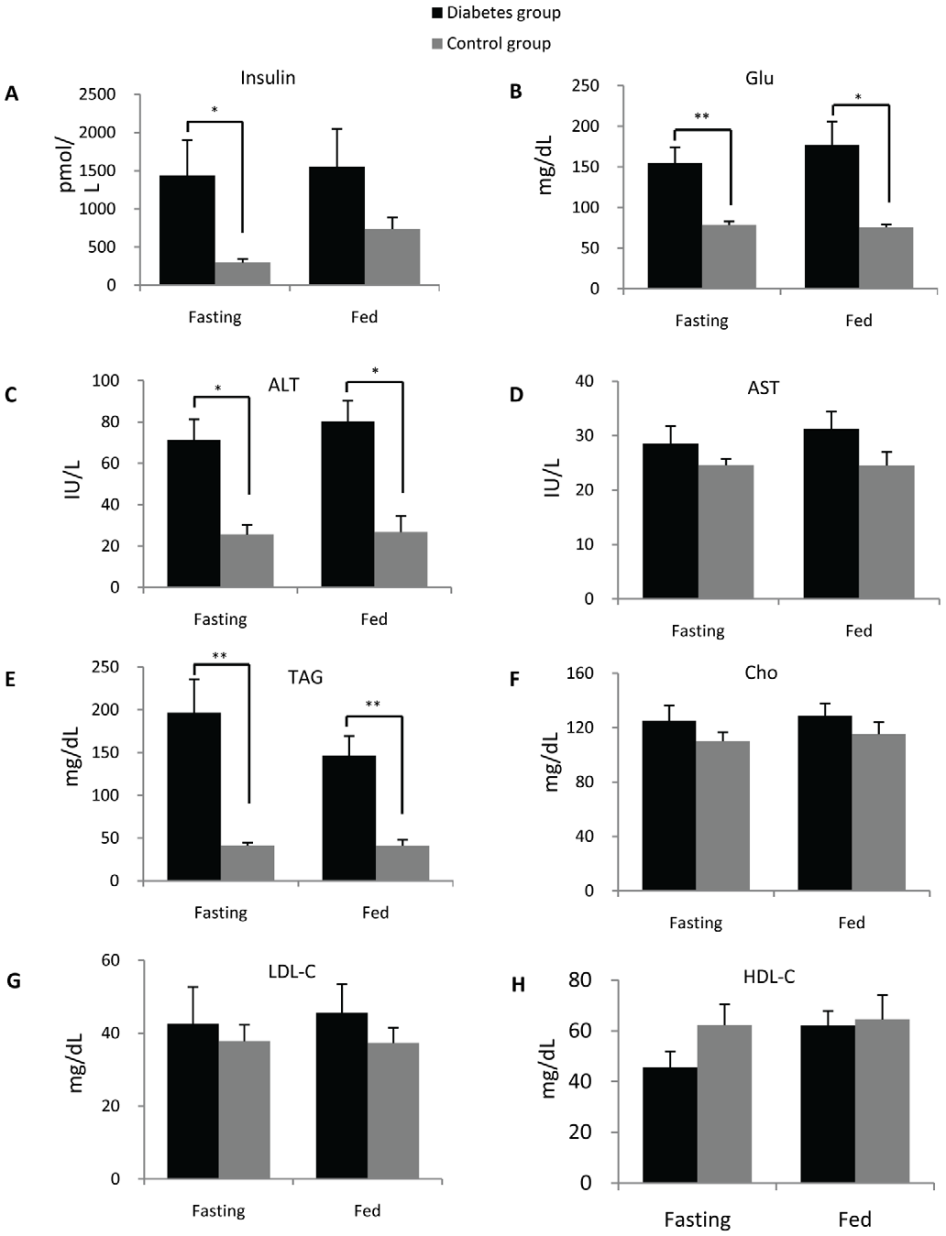
Biochemical analysis of plasma in diabetic monkeys (n = 8 for each group). (A) Insulin; (B) Glucose; (C) Alanine transaminase (ALT); (D) Aspartate transaminase (AST); (E) Triacylglycerides (TAG); (F) Cholesterol; (D) Low-density lipoprotein cholesterol (LDL-C); (H) High-density lipoprotein cholesterol (HDL-C). * p<0.05, ** p<0.005.

Diabetic monkeys demonstrated significantly higher levels of insulin, glucose and alanine transaminase (ALT) as well as slightly elevated levels of aspartate transaminase (AST) (Figure 4A–D). The differences in the levels of insulin and glucose under fasting condition were of greater significance than those acquired under normal chow-feeding condition (Figure 4A–B). Dramatic increases in plasma triacylglyceride levels in diabetic monkeys were also observed (Figure 4E), while there were no significant changes for the levels of cholesterol, HDL-cholesterol and LDL-cholesterol (Figure 4F–H).

**Figure 5:**
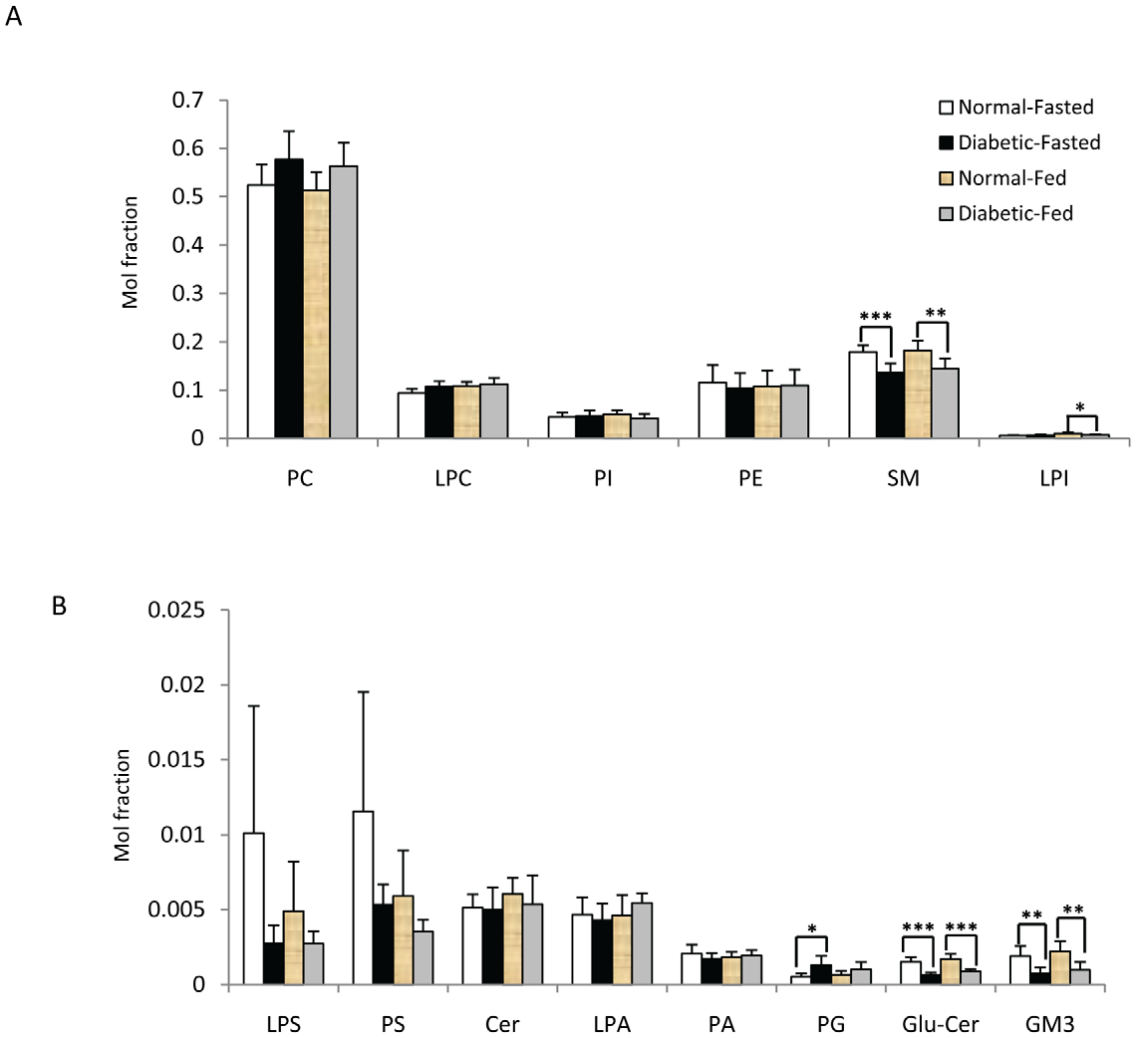
Comparison of total lipids in plasma of normal (n = 8) and diabetic (n = 8) monkey groups under both fasted and fed conditions. * p<0.05, ** p<0.005, *** p<0.0005.

**Figure 6:**
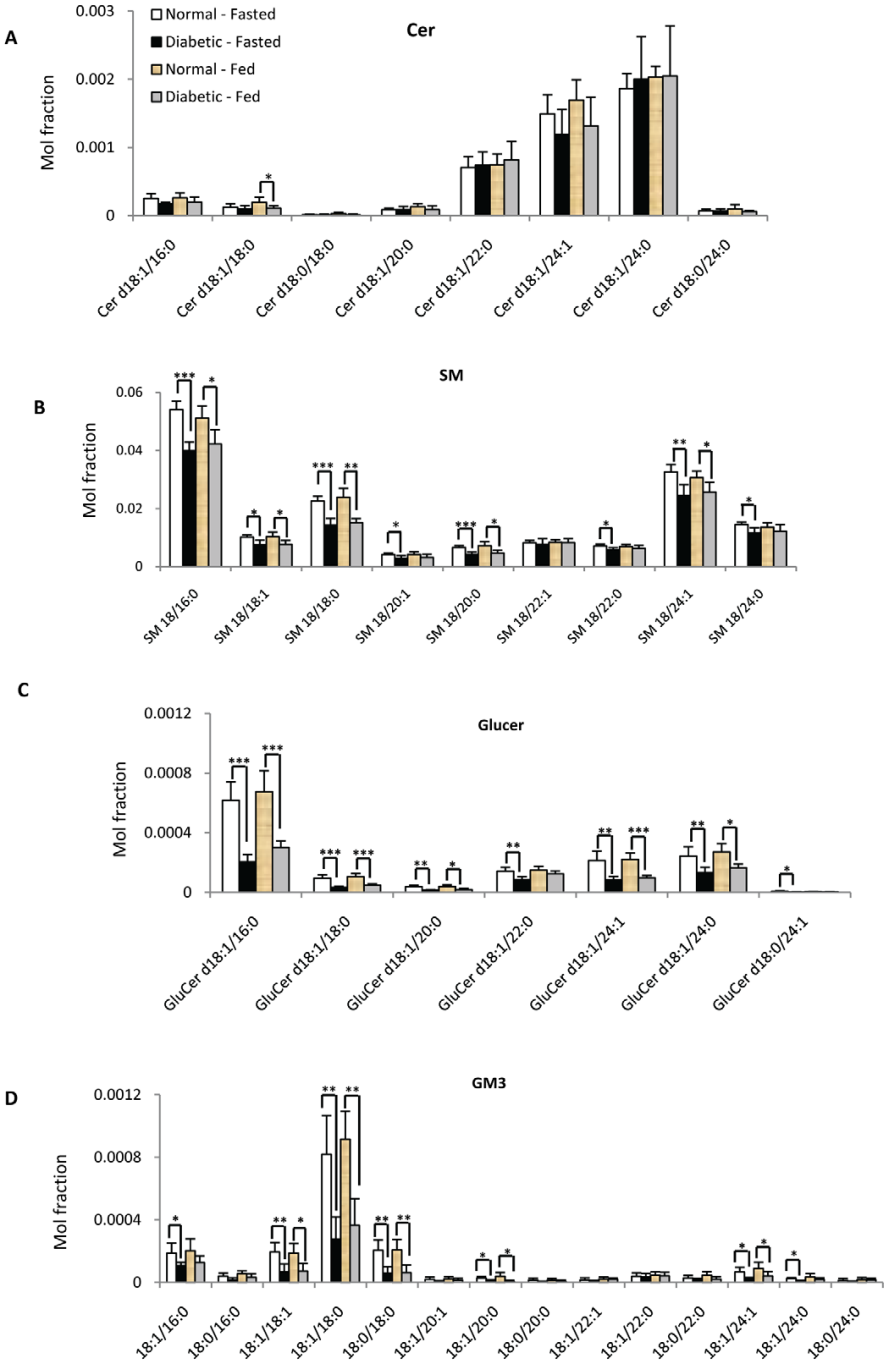
Individual sphingolipid species in plasma of normal (n = 8) and diabetic (n = 8) monkey under fasted and fed condition: (A) Cer, (B) SM (C), GluCer, (D) GM3. * p<0.05, ** p<0.005, *** p<0.0005.

**Figure 7:**
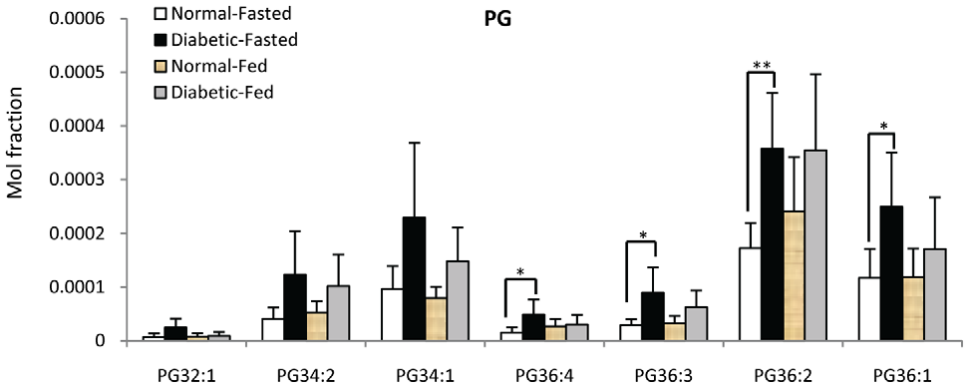
Comparison of individual PG species in normal (n = 8) and diabetic (n = 8) monkey under fasted and fed condition. * p<0.05, ** p<0.005, *** p<0.0005.

Crude extracts of plasma from normal and diabetic monkeys under fasting and fed conditions were examined by LCMS to quantitatively assess the differences in the levels of phospholipids and sphingolipids (Figure 5, 6, 7, Figure S2). The results showed that the supply of food did not cause considerable changes in plasma glycerophospholipids of normal or diabetic monkeys (Figure 5). We also compared the lipid profiles of diabetic monkeys at different ages; and lipidomic analysis revealed that there was no significant correlation between the levels of both glycerophospholipids and sphingolipids with age (12 to 20 years old). The diabetic monkeys were further stratified into two age groups and no appreciable changes in the quantities of the two lipid classes aforementioned were observed (Figure S3A, S3B). The maximum lifespan and age at sexual maturation of cynomolgus monkeys are 27 years and 3 years respectively [24]. Therefore, all monkeys (both normal controls and diabetic groups) used in the current study are considered sexually matured adults belonging to either the young (approximately 5 years) or middle-age categories (approximately 16 years), indicating that age was likely not a predominant factor for causing the observed changes in lipid levels between the diabetic and control groups in the current experimental design.

No significant changes were observed for most classes of glycerophospholipids (Figure 5A–B) except for PG, with the total PG level being significantly higher in diabetic monkeys compared to normal monkeys under fasting condition (Figure 5B). On the other hand, total LPI level was significantly lower in fed diabetic monkeys (Figure 5A).

The levels of total GM3, GluCer and SM were significantly lower in fasting diabetic monkeys than corresponding controls (Figure 5A, 5B). Moreover, detailed sphingolipid profiles indicated that a majority of the individual SM, GM3 and GluCer species followed the same general trend and were significantly decreased in the plasma of diabetic monkeys under both fed and fasting conditions compared to normal controls (Figure 6).

## Discussion

Plasma contains distinct lipid species yet the functions of many of these lipids remain poorly understood, particularly at the molecular level (Figure 1A). It has becoming increasingly clear that deregulated lipid metabolism plays an important role in many human diseases including metabolic syndrome diseases such as diabetes [25]. While the blood cholesterol and triacylglyceride levels of patients are diagnostic criteria regularly used by doctors for diabetes, information on other polar lipids, in particular sphingolipids and glycerophospholipids, is missing and gaining increasing interest from both researchers and doctors. Thus, monitoring the changes in various plasma polar lipids represents an emerging field in the treatment of metabolic syndrome and can possibly confer new perspectives in terms of disease diagnosis, molecular mechanisms triggering the disease and evaluation of treatment responses. Technological advancements, most notably in mass spectrometry, allow sensitive analysis of various lipids with diverse chemical composition. It is well-recognized now that glycerophospholipids and sphingolipids are well-ionized by electrospray. For instance, phosphatidylinositol (PI), phosphatidylethanolamine (PE), phosphatidylserine (PS), phosphatidylglycerol (PG), phosphatidic acid (PA) and gangliosides are better ionized in negative ESI mode [19]; while phosphatidylcholine (PC) ionizes much more efficiently in positive ESI mode. Most sphingolipids (Cer, GluCer and SM) can be detected in both positive and negative ESI modes.

In this study, we successfully separated and quantified the various polar lipids aforementioned using LCMS approaches [22]; and for the first time, we characterized and quantified the lipid profiles of monkey plasma and compared that with the corresponding lipid profiles in human plasma (Figure 1). In human plasma, our results indicated that PC and SM are most abundant polar lipids, accounting for 58% and 18% of the total polar lipids respectively, consistent with a recent report [26]. Lipidomic analysis revealed that PC and SM were the two most abundant polar lipids in monkey plasma as well (Figure 1B). Also, similar to the trend observed for human plasma, other major polar lipids, such as LPC, PE and PI, were shown to be present in proportionately much higher levels compared to the minor lipid species. The higher level of PUFA-containing lipid species found in monkey plasma could be partially attributed to a difference in diets, as monkeys are fed with a diet rich in seasonal fruits and vegetables [27]. Higher levels of plasmalogen species found in human plasma is likely associated with differences in the activities of enzymes involved in the biosynthesis of plasmalogens [28].

The similar profiles for polar lipids in human and monkey plasma might imply an analogous lipid biogenesis pathway that is conserved between human and cynomolgus monkeys. Furthermore, in a manner akin to human type 2 diabetes mellitus (T2DM), diabetic cynomolgus monkeys exhibited significantly higher plasma concentrations of TAG (Figure 4E) and marginally significant lower levels of HDL cholesterol (Figure 4F). Lipidomic analyses of TAG levels in diabetic monkeys also produced results consistent with that obtained using enzymatic colorimetric method (data not shown). These findings validated our approach in using cynomolgus monkeys as a model for the identification of potential lipid biomarkers that can possibly be applied to the diagnosis of the disease in humans.

For the first time, we used a specific LC-MRM approach with C17-GM3 ganglioside as an internal standard to quantify individual endogenous ganglioside GM3 species (Table 1), which are of significant value to both basic research as well as clinical investigations. For instance, decreases in ganglioside GM3 in Gne^M712T/M712T^ mouse muscle was recently reported to be potential marker for HIBM (Hereditary Inclusion Body Myopathy) diseases [29]. Furthermore, fingerprint of ganglioside GM3 can be potentially important as it is possible that changes in fatty acyl composition (saturation and length) in individual ganglioside GM3 species will affect its interaction with insulin receptor in membrane microdomains or the stability of the associated microdomains *per se*.

**Table 1:** MRM transitions of individual lipid species.

Lipid Species	MRM Transition	Lipid Species	MRM Transition	Lipid Species	MRM Transition
**PC- 34:2**	758→184	**PE- 36p:3**	724→196	**PA- 32:2**	643→153
**PC- 34:1**	760→184	**PE- 36p:2**	726→196	**PA- 32:1**	645→153
**PC- 36:4p**	766→184	**PE- 36p:1**	728→196	**PA- 32:0**	647→153
**PC- 36:3p**	768→184	**PE- 36:4**	738→196	**PA- 34:2**	671→153
**PC- 36:2p**	770→184	**PE- 36:3**	740→196	**PA- 34:1**	673→153
**PC- 36:1p**	772→184	**PE- 36:2**	742→196	**PA- 36:2**	699→153
**PC- 36:0p**	774→184	**PE- 36:1**	744→196	**PA- 36:1**	701→153
**PC- 36:5**	780→184	**PE- 38p:6**	746→196	**PA- 38:4**	723→153
**PC- 36:4**	782→184	**PE- 38p:5**	748→196	**LPA- 16:1**	407→153
**PC- 36:3**	784→184	**PE- 38p:4**	750→196	**LPA- 16:0**	409→153
**PC- 36:2**	786→184	**PE- 38:7**	760→196	**LPA- 18:1**	435→153
**PC- 36:1**	788→184	**PE- 38:6**	762→196	**LPA- 18:0**	437→153
**PC- 38:4p**	794→184	**PE- 38:5**	764→196		
**PC- 38:3p**	796→184	**PE- 38:4**	766→196	**GM3- 18:1/16:0**	1151→290
**PC- 38:2p**	798→184	**PE- 40p:6**	774→196	**GM3- 18:0/16:0**	1153→290
**PC- 38:6**	806→184	**PE- 40p:5**	776→196	**GM3- 18:1/18:1**	1177→290
**PC- 38:5**	808→184	**PE- 40p:4**	778→196	**GM3- 18:1/18:0**	1179→290
**PC- 38:4**	810→184	**PE- 40:6**	790→196	**GM3- 18:0/18:0**	1181→290
**PC- 38:3**	812→184	**PE- 40:5**	792→196	**GM3- 18:1/20:1**	1205→290
**PC- 40:4p**	822→184	**PE- 40:4**	794→196	**GM3- 18:1/20:0**	1207→290
**PC- 40:3p**	824→184	**PE- 42p:3**	808→196	**GM3- 18:0/20:0**	1209→290
**PC- 40:2p**	826→184	**PE- 42p:2**	810→196	**GM3- 18:1/22:1**	1233→290
**PC- 40:1p**	828→184	**PE- 42:9**	812→196	**GM3- 18:1/22:0**	1235→290
**PC- 40:7**	832→184	**PE- 42:8**	814→196	**GM3- 18:0/22:0**	1237→290
**PC- 40:6**	834→184	**PE- 42:7**	816→196	**GM3- 18:1/24:1**	1261→290
**PC- 40:5**	836→184			**GM3- 18:1/24:0**	1263→290
**LPC- 16e:0**	482→184	**PI- 32:1**	807→241	**GM3- 18:0/24:0**	1265→290
**LPC- 16:1**	494→184	**PI- 34:3**	831→241		
**LPC- 16:0**	496→184	**PI- 34:2**	833→241	**Cer- d18:1/16:0**	538→264
**LPC- 18:2**	520→/184	**PI- 34:1**	835→241	**Cer- d18:1/18:0**	566→264
**LPC- 18:1**	522→184	**PI- 36:4**	857→241	**Cer- d18:0/18:0**	568→266
**LPC- 18:0**	524→184	**PI- 36:3**	859→241	**Cer- d18:1/20:0**	594→264
**LPC- 20:0**	552→184	**PI- 36:2**	861→241	**Cer- d18:1/22:0**	622→264
		**PI- 36:1**	863→241	**Cer- d18:1/24:1**	648→264
**PS- 34:1**	760→673	**PI- 38:5**	883→24	**Cer- d18:1/24:0**	650→264
**PS- 36:2**	786→699	**PI- 38:4**	885→241	**Cer- d18:0/24:0**	652→266
**PS- 36:1**	788→701	**PI- 38:3**	887→241.		
**PS- 38:5**	808→721	**PI- 40:6**	909→241	**SM- 18/16:0**	703→184
**PS- 38:4**	810→723	**PI- 40:5**	911→241	**SM- 18/18:1**	729→184
**PS- 38:3**	812→725	**PI- 40:4**	913→24	**SM- 18/18:0**	731→184
**PS- 40:7**	832→745	**LPI- 16:1**	569→241	**SM- 18/20:1**	757→184
**PS- 40:6**	834→747	**LPI- 16:0**	571→241	**SM- 18/20:0**	759→184
**PS- 40:5**	836→749	**LPI- 18:1**	597→241	**SM- 18/22:1**	785→184
**LPS- 16:1**	494→407	**LPI- 18:0**	599→241	**SM- 18/22:0**	788→184
**LPS- 16:0**	496→409			**SM- 18/24:1**	813→184
**LPS- 18:1**	522→435	**PG- 32:1**	719→153	**SM- 18/24:0**	815→184
**LPS- 18:0**	524→437	**PG- 34:2**	745→153		
		**PG- 34:1**	747→153	**GluCer- d18:1/16:0**	700→264
**PE- 32:2**	686→196	**PG- 36:4**	769→153	**GluCer- d18:1/18:0**	728→264
**PE- 32:1**	688→196	**PG- 36:3**	771→153	**GluCer- d18:1/20:0**	756→264
**PE- 34p:2**	698→196	**PG- 36:2**	773→153	**GluCer- d18:1/22:0**	784→264
**PE- 34p:1**	700→196	**PG- 36:1**	775→153	**GluCer- d18:1/24:1**	810→264
**PE- 34:2**	714→196			**GluCer- d18:1/24:0**	812→264
**PE- 34:1**	716→196	**PA- 32:3**	641→153	**GluCer- d18:0/24:1**	812→266

Lipidomic data from this study showed that human plasma contained significantly higher levels of GluCer and ganglioside GM3 lipid species (Figure 1B), which are microdomain-associated lipids. We postulate that the levels of these lipids in other organs, such as the liver, would display a similar trend, with the level in human being considerably higher than that in monkey. However, this hypothesis requires further experimental verification.

Among all polar lipid species measured in this study, significant decreases in the levels of plasma GluCer, gangliosides GM3 and major SM species were observed in diabetic monkeys compared to normal controls, but not in the case of free ceramide species (Figure 6). The decrease in GluCer and ganglioside GM3 species in diabetic monkeys might be attributed to a down-regulation of glycosyltransferases activity, which transfers a specific sugar (e.g. glucose) from an appropriate sugar-nucleotide (e.g. UDP-Glc) to ceramide. This trend observed in diabetic monkeys was unexpected as GM3 has been reported as a negative regulator of insulin signaling [12], [30], [31]. Immediate analysis of diabetic human plasma samples needs to be carried out before considering glycosphingolipids as a potential therapeutic target in type 2 diabetes (ongoing work). Further studies will be required to validate the use of peripheral GluCer and gangliosides GM3 species as potential biomarkers for detecting the onset of diabetes in humans.

Interestingly, significant increases in the levels of plasma PG in fasting diabetic monkeys indicate that it could be potential biomarker (Figure 5B, Figure 7). PG is the precursor of cardiolipin, the key class of mitochondrial phospholipid that regulates mitochondrial function and oxidative stress [32], [33]. Multiple mechanisms have recently been proposed as the potential causes of insulin resistance and/or diabetes progression, and impaired mitochondrial function has emerged as a promising candidate that can possibly play a pathogenic role in the progression of diabetes [34], [35], [36], [37], [38], [39], [40]. Thus, further investigation on the polar lipid compositions including cardiolipin species in different organs of diabetic monkeys compared to normal controls will help to unravel these puzzles.

In addition, a decrease in the total level of LPI (Figure 5A) and marginally significant decreases in major PI species including PI38:4, 36:2, 36:3, 34:2 were also observed in diabetic monkeys under fed conditions (Figure S2). It should be noted, however, that such changes observed under fed conditions could be complicated by dietary factor and are thus less applicable to human clinical studies. Moreover, as similar changes were not observed under fasting conditions, such changes were unlikely to be a predominant result of the disease *per se*.

While there are no significant changes for the levels of most classes of phospholipids, alterations in the individual levels of certain phospholipid species were observed (Figure S1). For instance, there are no appreciable changes in the total level of PE in diabetic monkeys under both fed and fasted conditions when compared with the corresponding normal controls (Figure 5A), but the concentrations of most plasmalogen PE species such as pPE38:6, pPE38:5, pPE38:4 and pPE36:3 were significantly lower in diabetic monkeys under both fasting and fed conditions (Figure S2). Similar to the trends observed in PE species, diabetic monkeys demonstrated significantly higher levels for most diacyl PC species and decreased levels of plasmalogen PC species (Figure S1B). Plasmalogen refers to a specific class of phospholipids that contains a vinyl ether moiety at the sn-1 position of the glycerol backbone, making these lipids highly susceptible to oxidative damage as a result. The decreased levels of plasmalogen PE could possibly be attributed to intense lipid peroxidation induced by increased oxidative stress in diabetic monkeys or to a decrease in its biogenesis in the peroxisome [28].

A major drawback in the current study is the lack of age-matched controls due to the high cost implicated and substantial amount of logistic difficulties involved in the recruitment of healthy monkeys for academic investigation. In addition, the two monkey groups are also not gender-matched and have different body weight. Nevertheless, we did not observe significant changes for polar lipids among monkeys in the age range of 12 to 20 years, indicating that age was likely not a predominant factor for causing the observed changes in lipid levels in the diabetic monkeys. Another limitation in the current study design is the relatively small sample size (10 healthy human subjects vs 8 healthy monkeys; and 8 healthy monkeys vs 8 diabetic monkeys, respectively). Further studies using age-matched controls as well as a larger cohort size is essential to verify the preliminary findings obtained in the current study. Nevertheless, comparative lipidomic data between human plasma and monkey plasma could serve as a reference, and LC-MRM presented here provided other researchers an excellent to follow our approach to carry out related studies.

## Materials and Methods

### Chemicals

Chloroform and methanol were purchased from Merck (Merck Pte. Ltd., Singapore). Ammonium hydroxide (28% in H2O) was purchased from Sigma-Aldrich (St. Louis, MO, USA). Deionized water was obtained from a MilliQ purification system (Millipore, Bedford, MA, USA). PC-14:0/14:0, PE-14:0-14:0, PS-14:0/14:0, PA-17:0/17:0, PG-14:0/14:0, d31-PI-18:1/16:0, C17-Cer, C8-GucCer and C12-SM were obtained from Avanti Polar Lipids (Alabaster, AL, USA). Dioctanoyl phosphatidylinositol PI-8:0/8:0 was purchased from Echelon (Echelon Biosciences, Inc., Salt Lake City, UT, USA). C17-ganglioside GM3 was provided by Lilly.

### Monkey colony, selection and animal care

Sixteen cynomolgus monkeys, which were introduced from different regions of Southeast Asia, were selected from a colony in Guangdong Landau Biotech Co. (Guangdong, China) (Table S1). There is no inbreeding within this colony. Eight monkeys had developed hyperglycemia and hyperlipidemia spontaneously and were identified from the colony during the screening process. Eight other monkeys were normal and used as controls.

The animal welfare and steps taken to ameliorate suffering were in accordance with the recommendations of the Weatherall report on the use of non-human primates in research. Upon receiving the animals, a quarantine and acclimation period of approximately one month was allowed before any experiments were initiated in order to prevent the transmission of potentially contagious diseases and for the monkeys to adapt to the lab environment. Crown Bioscience Institutional Animal Care and Use Committee (IACUC) approved all animals procedures used in conducting these studies (Pri-dia-0901-010C). The monkeys were individually housed and maintained in accordance with the guidelines approved by the Association for Assessment and Accreditation of Laboratory Animal Care (AAALAC). All the procedures related to handling, care and treatment in this study were performed according to guidelines approved by the AAALAC.

Water was available ad libitum. All monkeys were fed twice daily with a complete, nutritionally balanced diet rich with seasonal fruits and vegetables.

### Collection of monkey plasma samples

Blood samples were collected from 8 diabetic and 8 normal control cynomolgus monkeys under fed and fasting conditions for biochemical and lipid analysis. All 16 monkeys were fasted overnight and 4 mL of blood was collected from a peripheral vein into an EDTA tube under ketamine (10 mg/kg IM injection) anesthesia. Blood samples under fed conditions were collected 2 days later. The monkeys were fed with normal chow (Shanghai shilin biotech Co., Ltd) at 8:00am following overnight fasting. Four mL of blood was collected from a peripheral vein into an EDTA tube at around one hour later under ketamine anesthesia.

Blood samples were kept on ice during plasma collection. The collected plasma was divided into 4 aliquots of 500 ul each and stored at −70°C. One aliquot was used for biochemical analysis, and remaining aliquots were used for LC-MS analysis of lipids.

### Collection of human plasma samples

Blood samples from 10 healthy non-fasting volunteers (Table S1) were collected in EDTA tubes at National University Hospital, Singapore. This study was approved by the institutional review board of National University of Singapore (04-115). All participants gave written informed consent for his study.

### Measurement of blood chemistry

Plasma total cholesterol (Cho) and triacylglycerides (TG) were measured using Cobas agents, Cholesterol-chod-pap and Triacylglycerides-GPO-PAP, respectively (Roche Diagnostics, Indianapolis, IN) by enzymatic colorimetric method. Glucose, alanine transaminase (ALT) and aspartate transaminase (AST) were measured using Cobas agents, Gluco-quant Glucose/HK, alanine transaminase acc. to IFCC with/without pyridoxal phosphate activation and aspartate transaminase acc. to IFCC with/without pyridoxal phosphate activation and (Roche Diagnostics), respectively, in UV test. High-density lipoprotein cholesterol (HDL) and low-density lipoprotein were measured using Cobas agents, HDL-cholesterol, no pretreatment, and LDL-cholesterol, no pretreatment, in homogeneous enzymatic colorimetric assay. Plasma insulin was assayed with Cobas Insulin agent in sandwich method. TC, TG, Glucose, ALT, AST, HDL and LDL were measured using a Clinical Analyzer Model 7600 (Hitach High-Technologies, Tokyo, Japan), and Insulin utilizing an E 170 Modules for MODULAR ANALYTICS (Roche Professional Diagnostics, Mannheim, Germany).

### Lipid extraction

Ice-cold solvent mixture (900 µl of chloroform∶methanol, 1∶2v/v) was added to 100 µl of plasma sample, vortexed, and incubated on ice in a vacuum chamber in a dark room for 1 h with agitation. After incubation, 0.3 ml of chloroform was added to the homogenate, followed by 0.35 ml of ice-cold water. The homogenate was then vortexed for 30 sec and centrifuged for 2 min at 9,000 rpm. The bottom organic phase was carefully transferred to an empty tube, and 0.5 ml of ice-cold chloroform was added for reextraction. The two organic extracts were then combined and dried under nitrogen. To ensure all lipids investigated were reasonable recovered, recovery of various lipids using in current extraction protocol were compared with a recent reported protocol [41], and comparable recoveries were obtained for all lipids investigated in this study.

### Quantitative analysis of lipids using HPLC/MS

Individual classes of polar lipids were separated using an Agilent 1200 HPLC system before introduction into a 3200 Q-Trap mass spectrometer (Applied Biosystems). The HPLC system contained an Agilent 1200 binary pump, an Agilent 1200 thermo sampler, and an Agilent 1200 column oven. HPLC conditions: Luna 3-µm silica column (i.d. 150×2.0 mm); mobile phase A (chloroform∶methanol∶ammonium hydroxide, 89.5∶10∶0.5), B (chloroform∶methanol∶ammonium hydroxide∶water, 55∶39∶0.5∶5.5); flow rate 300 µl/min; 5% B for 3 min, then linearly switched to 30% B in 24 min and maintained for 5 min, and then linearly changed to 70% B in 5 min and maintained for 7 min. Then, the composition of the mobile phase was returned to the original ratio] over 5 min and maintained for 6 min before the next sample was analyzed. Mass spectrometry was recorded under both positive and negative ESI modes with EMS scan type, and ESI conditions are: Turbo Spray source voltage, 5000 and −4500 volts for positive and negative, respectively; source temperature, 300°C; GS1: 40.00, GS2: 40.00, curtain gas: 25. PC, SM, Cer and GluCer are acquired in the positive ESI mode, while PE, PI, PS, PG, PA and GM3 are measured in in the negative ESI mode. Collision energies ranged from 20 to 65 volts for various lipid species in MS/MS scan modes. Multiple MRM transitions for individual GPL (PG, PE, PC, PI, PS and PA) species and SPL (SM, Cer, GluCer and GM3) were set up at different elution stages for LC-MS analysis [22], [23], [42]. Multiple reaction monitoring transitions of individual lipid species were listed in Table 1 (Table 1). Individual lipid species were quantified by comparison with spiked internal standards PC-14:0/14:0, PE-14:0-14:0, PS-14:0/14:0, PA-17:0/17:0, PG-14:0/14:0, d31-PI18:1/16:0, PI-8:0/8:0, C17-LPC, C17-LPA, C17-LPS, C17-Cer, C8-GluCer, C12-SM and C17-ganglioside GM3. The molar fractions of individual lipid species and each lipid class were normalization to summation of all polar lipid species aforementioned for comparison.

Neutral lipids were analyzed to verify data obtained using biochemical analysis aforementioned using HPLC/ESI/MS as described previously [43]. Briefly, triglycerides were separated from polar lipids on an Agilent Zorbax Eclipse XDB-C18 column (i.d. 4.6×150 mm), with chloroform∶methanol∶0.1 M ammonium acetate (100∶100∶4) as mobile phase at a flow rate of 0.25 mL. min^−1^. Selective ion monitoring was used to record major phospholipids, sterols and TAG species. TAGs were calculated as relative contents to the spiked d5-TAG 48∶0 internal standard (CDN isotopes) and were further normalized to total polar lipids.

### Statistics

Comparisons of the means of various plasma lipids were performed for human and monkey groups as well as for non-diabetic and diabetetic monkeys. Data are expressed as means with standard deviations as indicated by the error bars. The differences between human and monkey groups as well as for non-diabetic and diabetic monkeys were determined statistically using Student's t test, and a p-value<0.05 was considered statistically significant. The differences in individual lipid species between human and monkey groups, as well as between non-diabetic and diabetic monkeys were also evaluated using False Discovery Rate (FDR) adjustment. However, most of the q-values obtained from FDR adjustment were smaller than or approximately close to the corresponding p-values obtained using student t-test (Table S2, Table S3, Table S4). Thus, only p-values obtained using student t-test were presented in figures.

## Supporting Information

Figure S1 Plasma triacylglyceride (TAG) profiles in human (n = 10) and monkey (n = 8).
(PDF)

Figure S2 Individual glycerophospholipid species in plasma of normal and diabetic monkey under fasted (n = 8) and fed condition (n = 8). * p<0.05, ** p<0.005, *** p<0.0005.
(PDF)

Figure S3 Effects of aging on plasma lipid profiles of fasting diabetic monkeys. Scattered plotting for individual starved monkeys ranging from age 12 to 20; bar plotting shows non-significant changes between relatively young (12.75 year old) and old (18.75 year old) monkeys. (A): Phospholipids; (B): Sphingolipids.
(PDF)

Table S1 Age, body weight and gender of subjects used for this study.
(PDF)

Table S2 p-Value and false discovery rate (FDR) between human and monkey plasma lipids.
(PDF)

Table S3 p-Value and false discovery rate (FDR) between diabetic and healthy monkey plasma (fasting).
(PDF)

Table S4 p-Value and false discovery rate (FDR) between diabetic and healthy monkey plasma (fed).
(PDF)

## References

[1] Wild S, Roglic G, Green A, Sicree R, King H (2004). Global prevalence of diabetes: estimates for the year 2000 and projections for 2030.. Diabetes Care.

[2] Matheson A, Willcox MD, Flanagan J, Walsh BJ (2010). Urinary biomarkers involved in type 2 diabetes: a review.. Diabetes Metab Res Rev.

[3] Kahn SE, Hull RL, Utzschneider KM (2006). Mechanisms linking obesity to insulin resistance and type 2 diabetes.. Nature.

[4] Gross RW, Han X (2007). Lipidomics in diabetes and the metabolic syndrome.. Methods Enzymol.

[5] Holland WL, Brozinick JT, Wang LP, Hawkins ED, Sargent KM (2007). Inhibition of ceramide synthesis ameliorates glucocorticoid-, saturated-fat-, and obesity-induced insulin resistance.. Cell Metab.

[6] Aerts JM, Ottenhoff R, Powlson AS, Grefhorst A, van Eijk M (2007). Pharmacological inhibition of glucosylceramide synthase enhances insulin sensitivity.. Diabetes.

[7] Zhao H, Przybylska M, Wu IH, Zhang J, Siegel C (2007). Inhibiting glycosphingolipid synthesis improves glycemic control and insulin sensitivity in animal models of type 2 diabetes.. Diabetes.

[8] Pickersgill L, Litherland GJ, Greenberg AS, Walker M, Yeaman SJ (2007). Key role for ceramides in mediating insulin resistance in human muscle cells.. J Biol Chem.

[9] Chavez JA, Knotts TA, Wang LP, Li G, Dobrowsky RT (2003). A role for ceramide, but not diacylglycerol, in the antagonism of insulin signal transduction by saturated fatty acids.. J Biol Chem.

[10] Bijl N, Sokolovic M, Vrins C, Langeveld M, Moerland PD (2009). Modulation of glycosphingolipid metabolism significantly improves hepatic insulin sensitivity and reverses hepatic steatosis in mice.. Hepatology.

[11] van Eijk M, Aten J, Bijl N, Ottenhoff R, van Roomen CP (2009). Reducing glycosphingolipid content in adipose tissue of obese mice restores insulin sensitivity, adipogenesis and reduces inflammation.. PLoS One.

[12] Yamashita T, Hashiramoto A, Haluzik M, Mizukami H, Beck S (2003). Enhanced insulin sensitivity in mice lacking ganglioside GM3.. Proc Natl Acad Sci U S A.

[13] Langeveld M, Ghauharali KJ, Sauerwein HP, Ackermans MT, Groener JE (2008). Type I Gaucher disease, a glycosphingolipid storage disorder, is associated with insulin resistance.. J Clin Endocrinol Metab.

[14] Inokuchi J (2010). Membrane microdomains and insulin resistance.. FEBS Lett.

[15] Chen H, Dardik B, Qiu L, Ren X, Caplan SL (2010). Cevoglitazar, a novel peroxisome proliferator-activated receptor-alpha/gamma dual agonist, potently reduces food intake and body weight in obese mice and cynomolgus monkeys.. Endocrinology.

[16] Wagner JD, Shadoan MK, Zhang L, Ward GM, Royer LJ (2010). A selective peroxisome proliferator-activated receptor alpha agonist, CP-900691, improves plasma lipids, lipoproteins, and glycemic control in diabetic monkeys.. J Pharmacol Exp Ther.

[17] Kharitonenkov A, Wroblewski VJ, Koester A, Chen YF, Clutinger CK (2007). The metabolic state of diabetic monkeys is regulated by fibroblast growth factor-21.. Endocrinology.

[18] Han X, Gross RW (2005). Shotgun lipidomics: electrospray ionization mass spectrometric analysis and quantitation of cellular lipidomes directly from crude extracts of biological samples.. Mass Spectrom Rev.

[19] Shui G, Bendt AK, Pethe K, Dick T, Wenk MR (2007). Sensitive profiling of chemically diverse bioactive lipids.. J Lipid Res.

[20] Fei W, Shui G, Gaeta B, Du X, Kuerschner L (2008). Fld1p, a functional homologue of human seipin, regulates the size of lipid droplets in yeast.. J Cell Biol.

[21] Taguchi R, Houjou T, Nakanishi H, Yamazaki T, Ishida M (2005). Focused lipidomics by tandem mass spectrometry.. J Chromatogr B Analyt Technol Biomed Life Sci.

[22] Shui G, Guan XL, Gopalakrishnan P, Xue Y, Goh JS (2010). Characterization of substrate preference for Slc1p and Cst26p in Saccharomyces cerevisiae using lipidomic approaches and an LPAAT activity assay.. PLoS One.

[23] Chan R, Uchil PD, Jin J, Shui G, Ott DE (2008). Retroviruses human immunodeficiency virus and murine leukemia virus are enriched in phosphoinositides.. J Virol.

[24] Nakano M, Mizuno T, Gotoh S (1993). Accumulation of cardiac lipofuscin in crab-eating monkeys (Macaca fasicularis): the same rate of lipofuscin accumulation in several species of primates.. Mech Ageing Dev.

[25] Wenk MR (2005). The emerging field of lipidomics.. Nat Rev Drug Discov.

[26] Quehenberger O, Armando AM, Brown AH, Milne SB, Myers DS (2010). Lipidomics reveals a remarkable diversity of lipids in human plasma.. J Lipid Res.

[27] Toufektsian MC, Salen P, Laporte F, Tonelli C, de Lorgeril M (2011). Dietary flavonoids increase plasma very long-chain (n-3) fatty acids in rats.. J Nutr.

[28] Goldfine H (2010). The appearance, disappearance and reappearance of plasmalogens in evolution.. Prog Lipid Res.

[29] Paccalet T, Coulombe Z, Tremblay JP (2010). Ganglioside GM3 levels are altered in a mouse model of HIBM: GM3 as a cellular marker of the disease.. PLoS One.

[30] Kabayama K, Sato T, Saito K, Loberto N, Prinetti A (2007). Dissociation of the insulin receptor and caveolin-1 complex by ganglioside GM3 in the state of insulin resistance.. Proc Natl Acad Sci U S A.

[31] Tagami S, Inokuchi Ji J, Kabayama K, Yoshimura H, Kitamura F (2002). Ganglioside GM3 participates in the pathological conditions of insulin resistance.. J Biol Chem.

[32] Chen S, He Q, Greenberg ML (2008). Loss of tafazzin in yeast leads to increased oxidative stress during respiratory growth.. Mol Microbiol.

[33] Chicco AJ, Sparagna GC (2007). Role of cardiolipin alterations in mitochondrial dysfunction and disease.. Am J Physiol Cell Physiol.

[34] Patti ME, Corvera S (2010). The role of mitochondria in the pathogenesis of type 2 diabetes.. Endocr Rev.

[35] Anderson EJ, Lustig ME, Boyle KE, Woodlief TL, Kane DA (2009). Mitochondrial H2O2 emission and cellular redox state link excess fat intake to insulin resistance in both rodents and humans.. J Clin Invest.

[36] Mancuso DJ, Sims HF, Yang K, Kiebish MA, Su X (2010). Genetic ablation of calcium-independent phospholipase A2{gamma} prevents obesity and insulin resistance during high fat feeding by mitochondrial uncoupling and increased adipocyte fatty acid oxidation.. J Biol Chem.

[37] Choo HJ, Kim JH, Kwon OB, Lee CS, Mun JY (2006). Mitochondria are impaired in the adipocytes of type 2 diabetic mice.. Diabetologia.

[38] Okamoto Y, Higashiyama H, Rong JX, McVey MJ, Kinoshita M (2007). Comparison of mitochondrial and macrophage content between subcutaneous and visceral fat in db/db mice.. Exp Mol Pathol.

[39] Rong JX, Qiu Y, Hansen MK, Zhu L, Zhang V (2007). Adipose mitochondrial biogenesis is suppressed in db/db and high-fat diet-fed mice and improved by rosiglitazone.. Diabetes.

[40] Maasen JA (2008). Mitochondria, body fat and type 2 diabetes: what is the connection?. Minerva Med.

[41] Zhao Z, Xu Y (2010). An extremely simple method for extraction of lysophospholipids and phospholipids from blood samples.. J Lipid Res.

[42] Guan XL, He X, Ong WY, Yeo WK, Shui G (2006). Non-targeted profiling of lipids during kainate-induced neuronal injury.. FASEB J.

[43] Shui G, Guan XL, Low CP, Chua GH, Goh JS (2010). Toward one step analysis of cellular lipidomes using liquid chromatography coupled with mass spectrometry: application to Saccharomyces cerevisiae and Schizosaccharomyces pombe lipidomics.. Mol Biosyst.

