# Supplementary figures and images for: Comparative Plasma Lipidome between Human and Cynomolgus Monkey: Are Plasma Polar Lipids Good Biomarkers for Diabetic Monkeys?
Source: PLoS One. 2011 May 4;6(5):e19731. doi: 10.1371/journal.pone.0019731 (PMC3087804; doi:10.1371/journal.pone.0019731)

Figure S1

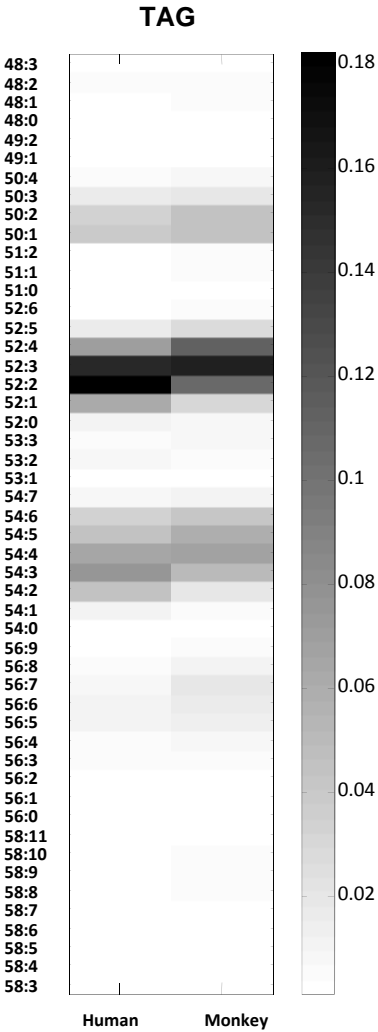

Supplement: Figure S1 — Plasma triacylglyceride (TAG) profiles in human (n = 10) and monkey (n = 8). (PDF) [file pone.0019731.s001.pdf]

Figure S2

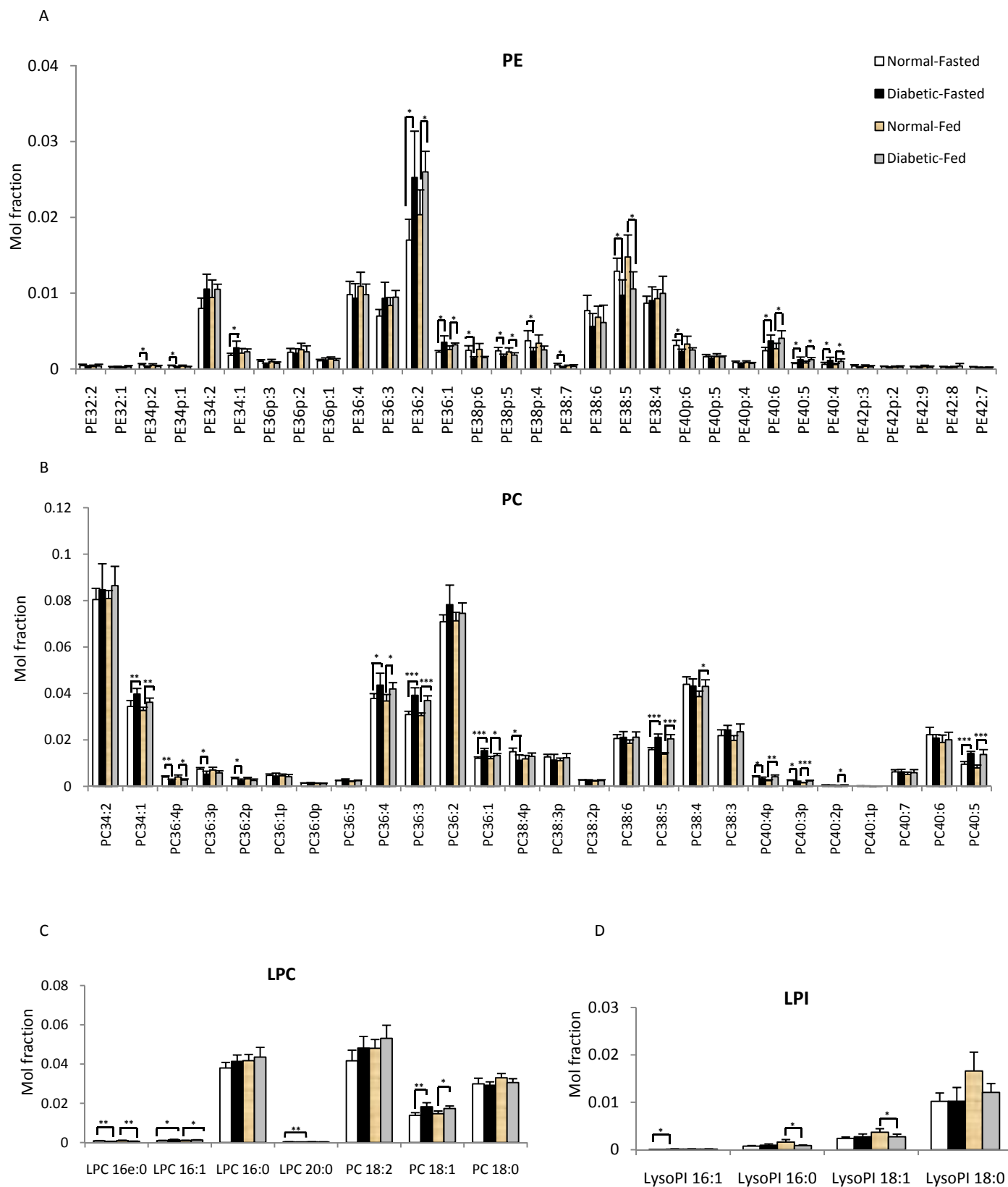

**Figure S2**

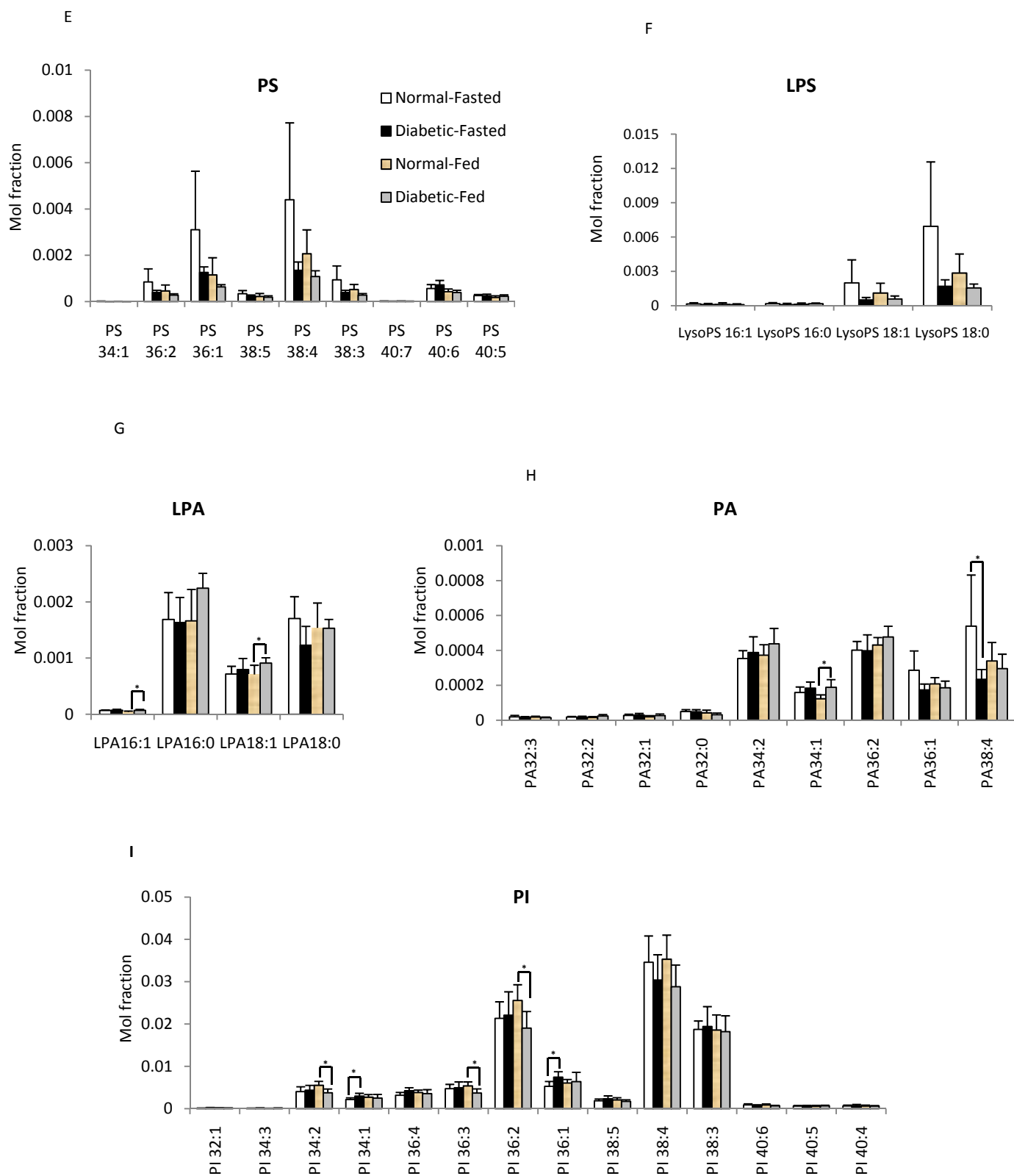

Supplement: Figure S2 — Individual glycerophospholipid species in plasma of normal and diabetic monkey under fasted (n = 8) and fed condition (n = 8). * p<0.05, ** p<0.005, *** p<0.0005. (PDF) [file pone.0019731.s002.pdf]

**Figure S3**

**A**

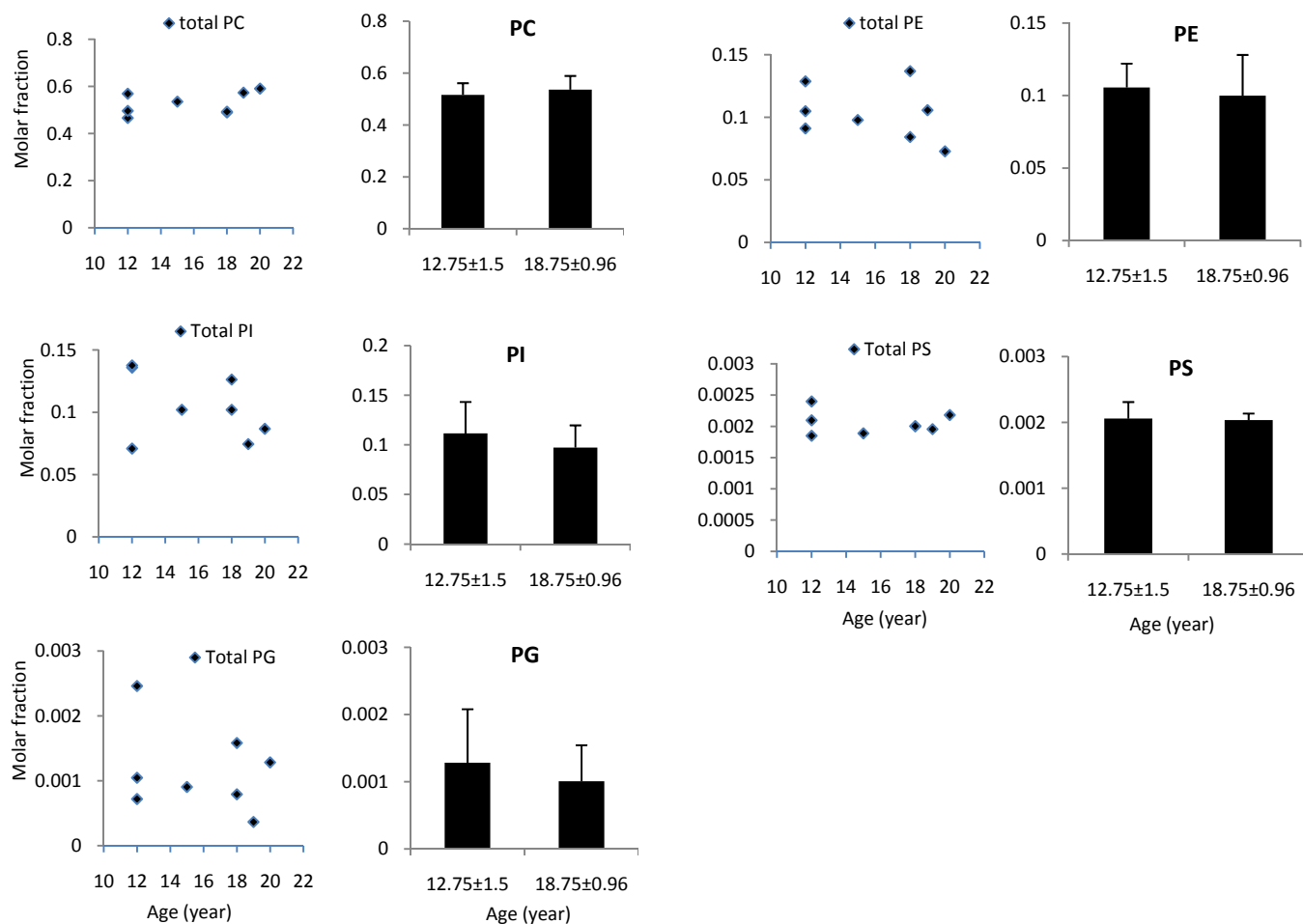

**B**

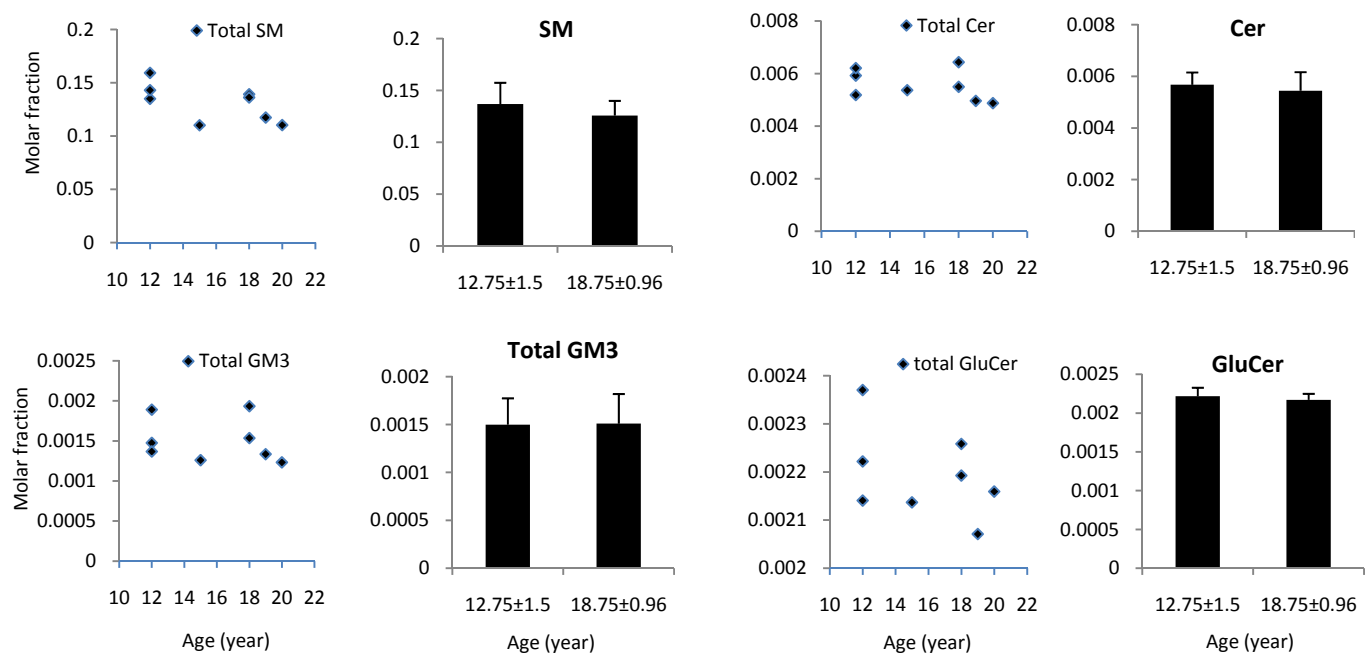

Supplement: Figure S3 — Effects of aging on plasma lipid profiles of fasting diabetic monkeys. Scattered plotting for individual starved monkeys ranging from age 12 to 20; bar plotting shows non-significant changes between relatively young (12.75 year old) and old (18.75 year old) monkeys. (A): Phospholipids; (B): Sphingolipids. (PDF) [file pone.0019731.s003.pdf]
