# Supplementary material for: Comparative Plasma Lipidome between Human and Cynomolgus Monkey: Are Plasma Polar Lipids Good Biomarkers for Diabetic Monkeys?
Source: PLoS One. 2011 May 4;6(5):e19731. doi: 10.1371/journal.pone.0019731 (PMC3087804; doi:10.1371/journal.pone.0019731)
Supplement: Table S1 — Age, body weight and gender of subjects used for this study. (PDF) [file pone.0019731.s004.pdf]

Table S1

	Normal monkey (n=8)	Diabetic monkey (n=8)	Healthy human volunteers (n=10)
Age	5.1±0.4	15.8±3.0	39.5±11.5
Body weight	4.9±0.4	8.5±2.1	67.6±12.4
Gender	8M	5F, 3M	5F, 5M

M, male; F, female. Values are mean ± standard deviation
