# Supplementary material for: Comparative Plasma Lipidome between Human and Cynomolgus Monkey: Are Plasma Polar Lipids Good Biomarkers for Diabetic Monkeys?
Source: PLoS One. 2011 May 4;6(5):e19731. doi: 10.1371/journal.pone.0019731 (PMC3087804; doi:10.1371/journal.pone.0019731)
Supplement: Table S2 — p-Value and false discovery rate (FDR) between human and monkey plasma lipids. (PDF) [file pone.0019731.s005.pdf]

Table S2 p-Value and false discovery rate (FDR) between human and monkey plasma lipids

	Student T-TEST	FDR		Student T-TEST	FDR
PE32:2	0.06244449	0.011741699	PI 32:1	0.60131203	0.091233549
PE32:1	0.042680072	0.008383586	PI 34:3	0.214936508	0.035553407
PE34p:2	0.007017544	0.001754386	PI 34:2	0.018193682	0.004084296
PE34p:1	0.003582239	0.000961089	PI 34:1	0.047509097	0.009168422
PE34:2	0.003062462	0.00083178	PI 36:4	0.00099892	0.000309525
PE34:1	0.02111043	0.00455323	PI 36:3	0.004757231	0.001245941
PE36p:3	0.002622527	0.000730324	PI 36:2	0.01001731	0.002319798
PE36p:2	2.52134E-05	1.45972E-05	PI 36:1	0.484689762	0.07509278
PE36p:1	0.038695222	0.007739044	PI 38:5	0.366072339	0.057939507
PE36:4	0.000535395	0.000181211	PI 38:4	0.001592075	0.00047332
PE36:3	5.0297E-05	2.57334E-05	PI 38:3	0.000100549	4.51444E-05
PE36:2	0.006445454	0.001668235	PI 40:6	0.000842788	0.000272667
PE36:1	0.000180717	7.22868E-05	PI 40:5	0.03404033	0.006934141
PE38p:6	0.652170759	0.098272306	PI 40:4	0.045768872	0.008910754
PE38p:5	7.73549E-05	3.69958E-05	LysoPS 16:1	0.391003847	0.061443462
PE38p:4	0.001140596	0.000348515	LysoPS 16:0	0.990709728	0.142454994
PE38:7	0.000230587	8.74639E-05	LysoPS 18:1	0.079151519	0.014391185
PE38:6	0.004430878	0.001174449	LysoPS 18:0	0.048012756	0.009185049
PE38:5	0.000118599	5.01766E-05	PS 34:1	0.245735363	0.040045763
PE38:4	0.099360329	0.017628446	PS 36:2	0.02228826	0.004760599
PE40p:6	0.000248291	9.25832E-05	PS 36:1	0.03489882	0.007043799
PE40p:5	0.440477301	0.068726955	PS 38:5	0.010909392	0.002500069
PE40p:4	0.102896298	0.01796602	PS 38:4	0.012018712	0.002725893
PE40:6	0.103141162	0.017866973	PS 38:3	0.006903602	0.001745738
PE40:5	0.000429775	0.000157584	PS 40:7	0.009294725	0.002222652
PE40:4	0.000161765	6.59042E-05	PS 40:6	9.42312E-05	4.31893E-05
PE42p:3	0.060199577	0.011417161	PS 40:5	0.001240958	0.000373987
PE42p:2	0.075401795	0.013823662	PG32:1	0.000112823	4.86688E-05
PE42:9	0.806284045	0.118254993	PG34:2	0.008913552	0.002154925
PE42:8	0.097952512	0.017519961	PG34:1	0.074248142	0.013726547
PE42:7	0.319154463	0.051251082	PG36:4	0.10007113	0.017612519
LysoPC16e:0	0.018625108	0.004138913	PG36:3	0.023925845	0.005013034
LysoPC 16:1	0.033129194	0.00681161	PG36:2	0.002980261	0.000819572
Lyso PC 16:0	1.71918E-07	1.99063E-07	PG36:1	0.018921989	0.004162837
Lyso PC 18:2	1.73961E-07	1.91357E-07	PA32:3	0.000851923	0.000271628
LysoPC 18:1	0.137960446	0.023347152	PA32:2	0.000860889	0.000270565
LysoPC 18:0	0.338170846	0.053911294	PA32:1	0.002544248	0.000717608
LysoPC20:0	0.274965615	0.044479732	PA32:0	0.009604177	0.002247786
SM18/16:0	4.56658E-05	2.51162E-05	PA34:2	2.62244E-06	2.06049E-06
SM18/18:1	0.174801354	0.029133559	PA34:1	2.93679E-05	1.65665E-05
SM18/18:0	0.080747877	0.014561093	PA36:2	1.35E-07	1.64999E-07
SM18/20:1	0.041373363	0.008200126	PA36:1	5.68464E-06	4.16874E-06
SM18/20:0	1.67861E-06	1.36775E-06	PA38:4	0.000487279	0.000170161
SM18/22:1	6.2869E-06	4.46167E-06	LPA16:1	0.530944615	0.081683787
SM18/22:0	2.40449E-07	2.51899E-07	LPA16:0	0.107335951	0.018448367
SM18/24:1	0.000191374	7.51826E-05	LPA18:1	0.120285004	0.020513722
SM18/24:0	0.821393472	0.119673221	LPA18:0	0.065636747	0.01223736
PC34:2	0.007474822	0.001847709	GM3 18:1/16:0	7.14812E-06	4.62525E-06
PC34:1	5.79049E-09	1.06159E-08	GM3 18:0/16:0	1.0448E-06	9.19423E-07
PC36:4p	0.020259485	0.004412957	GM3 18:1/18:1	0.002004791	0.000588072
PC36:3p	0.000497013	0.000170848	GM3 18:1/18:0	0.850235934	0.123060464
PC36:2p	0.002333116	0.000666605	GM3 18:0/18:0	0.798877009	0.118751988
PC36:1p	0.009503018	0.002248026	GM3 18:1/20:1	1.71528E-05	1.01989E-05
PC36:0p	2.56138E-13	8.05006E-13	GM3 18:1/20:0	4.78733E-05	2.50765E-05
PC36:5	6.30302E-16	3.46666E-15	GM3 18:0/20:0	1.02103E-06	9.35945E-07
PC36:4	9.46246E-09	1.60134E-08	GM3 18:1/22:1	1.26049E-05	7.703E-06
PC36:3	3.32885E-15	1.46469E-14	GM3 18:1/22:0	1.00949E-05	6.34536E-06
PC36:2	6.99979E-06	4.66653E-06	GM3 18:0/22:0	9.16829E-05	4.29154E-05
PC36:1	0.713590307	0.106795828	GM3 18:1/24:1	1.77822E-08	2.60806E-08
PC38:4p	7.99604E-08	1.03478E-07	GM3 18:1/24:0	1.46423E-06	1.23896E-06
PC38:3p	6.63176E-06	4.55933E-06	GM3 18:0/24:0	3.58434E-07	3.58434E-07
PC38:2p	1.84087E-18	2.02496E-17	Cer d18:1/16:0	0.803690025	0.118665641
PC38:6	6.21573E-05	3.10786E-05	Cer d18:1/18:0	0.149224727	0.025060641
PC38:5	1.55634E-10	4.27993E-10	Cer d18:0/18:0	0.007549298	0.001845384
PC38:4	0.590766693	0.090256023	Cer d18:1/20:0	0.023155237	0.004898223
PC38:3	0.000439879	0.000156086	Cer d18:1/22:0	0.026134128	0.005424064
PC40:4p	3.61564E-07	3.45844E-07	Cer d18:1/24:1	0.000108983	4.79526E-05
PC40:3p	3.72785E-15	1.36688E-14	Cer d18:1/24:0	7.62823E-08	1.04888E-07
PC40:2p	1.48667E-18	3.27068E-17	Cer d18:0/24:0	0.221994348	0.036446833
PC40:1p	3.32047E-16	2.43501E-15	GluCer d18:1/16:0	0.000225358	8.69802E-05
PC40:7	0.000759928	0.000249529	GluCer d18:1/18:0	6.45457E-05	3.15557E-05
PC40:6	0.00014526	6.02967E-05	GluCer d18:1/20:0	5.40095E-10	1.32023E-09
PC40:5	0.000607098	0.000202366	GluCer d18:1/22:0	2.32303E-09	4.64606E-09
LysoPI 16:1	0.000436776	0.000157526	GluCer d18:1/24:1	1.72837E-08	2.716E-08
LysoPI 16:0	0.002247243	0.000650518	GluCer d18:1/24:0	7.33067E-10	1.61275E-09
LysoPI 18:1	4.7337E-05	2.54004E-05	GluCer d18:0/24:1	2.9949E-06	2.27199E-06
LysoPI 18:0	0.006720444	0.001719183			
