# Supplementary material for: Comparative Plasma Lipidome between Human and Cynomolgus Monkey: Are Plasma Polar Lipids Good Biomarkers for Diabetic Monkeys?
Source: PLoS One. 2011 May 4;6(5):e19731. doi: 10.1371/journal.pone.0019731 (PMC3087804; doi:10.1371/journal.pone.0019731)
Supplement: Table S3 — p-Value and false discovery rate (FDR) between diabetic and healthy monkey plasma (fasting). (PDF) [file pone.0019731.s006.pdf]

Table S3. p-Value and false discovery rate (FDR) between diabetic and healthy monkey plasma (fasting)

	Student T-TEST	FDR		Student T-TEST	FDR
Cer d18:1/16:0	0.039477968	0.035226494	LysoPS 16:1	0.508124004	0.235769538
Cer d18:1/18:0	0.448338094	0.218517727	LysoPS 16:0	0.331975049	0.178282897
Cer d18:0/18:1	0.794586487	0.326851179	LysoPS 18:1	0.132403777	0.084389221
Cer d18:1/20:0	0.928453374	0.366328542	LysoPS 18:0	0.053592498	0.042004931
Cer d18:1/22:0	0.650333803	0.283604215	PS 34:1	0.040875269	0.035920691
Cer d18:1/24:1	0.211510477	0.123915229	PS 36:2	0.109995487	0.071682452
Cer d18:1/24:0	0.534126365	0.242026009	PS 36:1	0.182739943	0.111567544
Cer d18:0/24:0	0.975137711	0.372092021	PS 38:5	0.171662264	0.105919269
GluCer d18:1/16:0	3.15786E-05	0.000183156	PS 38:4	0.066084838	0.047911507
GluCer d18:1/18:0	4.7615E-05	0.000212436	PS 38:3	0.081497785	0.056950259
GluCer d18:1/20:0	0.000721957	0.002093675	PS 40:7	0.405848891	0.202924446
GluCer d18:1/22:0	0.0017423	0.003484599	PS 40:6	0.224923123	0.125437896
GluCer d18:1/24:1	0.001480235	0.003302062	PS 40:5	0.970470537	0.375248608
GluCer d18:1/24:0	0.003346092	0.005544952	PE32:2	0.205239884	0.121468503
GluCer d18:0/24:1	0.034001509	0.031807863	PE32:1	0.484459825	0.226602176
SM 18/16:0	1.45875E-05	0.000141012	PE34p:2	0.368454781	0.194276157
SM 18/18:1	0.012741059	0.017594796	PE34p:1	0.454503265	0.219676578
SM 18/18:0	3.03588E-05	0.000195645	PE34:2	0.385097332	0.195926713
SM 18/20:1	0.024817336	0.026171009	PE34:1	0.056138908	0.043414089
SM 18/20:0	0.000232867	0.000750348	PE36p:3	0.000126051	0.000430055
SM 18/22:1	0.728851718	0.306328983	PE36p:2	0.061065423	0.045997332
SM 18/22:0	0.013655884	0.018419564	PE36p:1	0.002658954	0.004673313
SM 18/24:1	0.002683544	0.00457781	PE36:4	1.20994E-05	0.000140353
SM 18/24:0	0.015050806	0.018977103	PE36:3	0.714318295	0.302412125
GM3 18:1/16:0	0.034236187	0.031519029	PE36:2	4.7311E-05	0.00022867
GM3 18:0/16:0	0.103728699	0.069152466	PE36:1	9.29502E-05	0.000336944
GM3 18:1/18:1	0.004043041	0.00633774	PE38p:6	0.000632251	0.00193003
GM3 18:1/18:0	0.001215247	0.002819373	PE38p:5	0.136107591	0.085806959
GM3 18:0/18:0	0.001919154	0.003710365	PE38p:4	0.470921111	0.222060361
GM3 18:1/20:1	0.062939681	0.04620888	PE38:7	6.32803E-05	0.000262161
GM3 18:1/20:0	0.024224752	0.026019178	PE38:6	8.8085E-06	0.000127723
GM3 18:0/20:0	0.093347112	0.062955029	PE38:5	0.010830686	0.015321459
GM3 18:1/22:1	0.293180428	0.16041948	PE38:4	1.76664E-05	0.000146379
GM3 18:1/22:0	0.835646834	0.338933681	PE40p:6	1.86946E-06	5.42144E-05
GM3 18:0/22:0	0.1507672	0.094026856	PE40p:5	0.001191775	0.002880122
GM3 18:1/24:1	0.013865184	0.018276833	PE40p:4	0.001699121	0.003519608
GM3 18:1/24:0	0.0068709	0.010487163	PE40:6	6.47499E-05	0.000250366
GM3 18:0/24:0	0.242920266	0.134184528	PE40:5	0.000735493	0.002031362
PG32:1	0.047804289	0.03850901	PE40:6	0.111601425	0.071920918
PG34:2	0.062876541	0.046754351	PE42p:3	0.021833754	0.024830543
PG34:1	0.04309326	0.037304613	PE42p:2	0.016433602	0.019857269
PG36:4	0.030682622	0.031220913	PE42:9	0.445023777	0.2187405
PG36:3	0.024110072	0.026384607	PE42:8	0.187059928	0.113015373
PG36:2	0.003347775	0.005393638	PE42:7	0.213477131	0.122590828
PG36:1	0.029923287	0.030991976	LPC16e:0	0.002220597	0.004024832
LPA16:1	0.668172162	0.289208846	LPC16:1	0.008556095	0.012406338
LPA16:0	0.974123233	0.37416654	LPC16:0	0.05798367	0.044250695
LPA18:1	0.37877812	0.194417088	LPC18:2	0.044060283	0.03758083
LPA18:0	0.087933515	0.060715998	LPC18:1	0.001145417	0.002888443
LysoPI 16:1	0.031861701	0.031321672	LPC18:0	0.901040611	0.35794764
LysoPI 16:0	0.106189413	0.069988477	LPC20:0	0.001081914	0.002852319
LysoPI 18:1	0.20401664	0.121989331	PC34:2	0.212255652	0.123108278
LysoPI 18:0	0.871214862	0.348485945	PC34:1	0.001687886	0.00362583
PI 32:1	0.080460803	0.0569113	PC36:4p	0.001982825	0.003709801
PI 34:3	0.044405447	0.036793085	PC36:3p	0.007179827	0.010677691
PI 34:2	0.604269695	0.27168715	PC36:2p	0.033559519	0.031909051
PI 34:1	0.047208762	0.038564904	PC36:1p	0.733088254	0.305892941
PI 36:4	0.031477069	0.031477069	PC36:0p	0.608807114	0.271621635
PI 36:3	0.67703017	0.290872221	PC36:5	0.024005473	0.026775335
PI 36:2	0.696049563	0.296844667	PC36:4	0.015524774	0.019158232
PI 36:1	0.019387332	0.022948271	PC36:3	4.13178E-05	0.000217858
PI 38:5	0.221167217	0.125761751	PC36:2	0.021601671	0.025057938
PI 38:4	0.456501435	0.21881887	PC36:1	6.33316E-07	3.67323E-05
PI 38:3	0.62612994	0.275117701	PC38:4p	0.014465948	0.018644999
PI 40:6	0.328438764	0.178032227	PC38:3p	0.368880335	0.192748283
PI 40:5	0.468221312	0.222597017	PC38:2p	0.372850938	0.193083522
PI 40:4	0.531094721	0.242547195	PC38:6	0.406995834	0.201758619
PA32:3	0.401391436	0.202440898	PC38:5	4.68441E-06	9.05653E-05
PA32:2	0.949751357	0.372199856	PC38:4	0.822106095	0.335789814
PA32:1	0.524214431	0.241305056	PC38:3	0.044342829	0.037273682
PA32:0	0.965099037	0.375676135	PC40:4p	0.06937839	0.049678353
PA34:2	0.351270352	0.186914499	PC40:3p	0.034459285	0.031228727
PA34:1	0.222066464	0.125047135	PC40:2p	0.088879602	0.060647258
PA36:2	0.853875753	0.343922178	PC40:1p	0.98059717	0.371729646
PA36:1	0.050169978	0.039861078	PC40:7	0.756578836	0.313439803
PA38:4	0.033197928	0.03209133	PC40:6	0.616500833	0.272954567
			PC40:5	2.27141E-05	0.000164677
