# Supplementary material for: Comparative Plasma Lipidome between Human and Cynomolgus Monkey: Are Plasma Polar Lipids Good Biomarkers for Diabetic Monkeys?
Source: PLoS One. 2011 May 4;6(5):e19731. doi: 10.1371/journal.pone.0019731 (PMC3087804; doi:10.1371/journal.pone.0019731)
Supplement: Table S4 — p-Value and false discovery rate (FDR) between diabetic and healthy monkey plasma (fed). (PDF) [file pone.0019731.s007.pdf]

Table S4. p-Value and false discovery rate (FDR) between diabetic and healthy monkey plasma (fed)

	Student T-TEST	FDR		Student T-TEST	FDR
Cer d18:1/16:0	0.208759069	0.13325047	LysoPS 16:1	0.76080797	0.323748072
Cer d18:1/18:0	0.034952082	0.044619679	LysoPS 16:0	0.488128779	0.238111599
Cer d18:0/18:1	0.081058969	0.07152262	LysoPS 18:1	0.215145164	0.134465728
Cer d18:1/20:0	0.174689538	0.115179915	LysoPS 18:0	0.172380565	0.116211617
Cer d18:1/22:0	0.666225848	0.293923168	PS 34:1	0.171816044	0.117147303
Cer d18:1/24:1	0.128920965	0.092086403	PS 36:2	0.234347972	0.144957509
Cer d18:1/24:0	0.99351324	0.389613035	PS 36:1	0.237633672	0.145490003
Cer d18:0/24:0	0.199569782	0.128754698	PS 38:5	0.572334146	0.268281631
GluCer d18:1/16:0	5.58216E-05	0.000478471	PS 38:4	0.118248885	0.087591767
GluCer d18:1/18:0	9.83407E-05	0.000590044	PS 38:3	0.081559141	0.070920992
GluCer d18:1/20:0	0.006181677	0.014265409	PS 40:7	0.173485442	0.115656961
GluCer d18:1/22:0	0.112566406	0.085493473	PS 40:6	0.510016353	0.246782106
GluCer d18:1/24:1	7.07662E-05	0.000471774	PS 40:5	0.274960469	0.155638001
GluCer d18:1/24:0	0.003130608	0.008538021	PE32:2	0.659030876	0.292902612
GluCer d18:0/24:1	0.124769835	0.091295001	PE32:1	0.168792459	0.116408592
SM 18/16:0	0.006107108	0.014657059	PE34p:2	0.862596338	0.349701218
SM 18/18:1	0.013559193	0.023927987	PE34p:1	0.941173996	0.371516051
SM 18/18:0	0.000238164	0.000893116	PE34:2	0.103696352	0.082957081
SM 18/20:1	0.125804604	0.090943087	PE34:1	0.372449983	0.190999991
SM 18/20:0	0.009037076	0.017491115	PE36p:3	1.48109E-05	0.000296219
SM 18/22:1	0.81394348	0.336804199	PE36p:2	0.011456607	0.020830195
SM 18/22:0	0.269525748	0.155495624	PE36p:1	0.005062918	0.013207612
SM 18/24:1	0.010510845	0.019707833	PE36:4	0.000183996	0.000735983
SM 18/24:0	0.249310875	0.1510975	PE36:3	0.523888242	0.251466356
GM3 18:1/16:0	0.07210653	0.068672886	PE36:2	0.000132185	0.000721007
GM3 18:0/16:0	0.09663899	0.079429307	PE36:1	2.74533E-05	0.00032944
GM3 18:1/18:1	0.005468983	0.013672457	PE38p:6	0.020025104	0.02730696
GM3 18:1/18:0	0.000831603	0.00293507	PE38p:5	0.585925948	0.27042736
GM3 18:0/18:0	0.002900197	0.008286277	PE38p:4	0.066329189	0.071066988
GM3 18:1/20:1	0.443145972	0.221572986	PE38:7	2.09692E-05	0.000314538
GM3 18:1/20:0	0.013959184	0.023930031	PE38:6	5.39618E-05	0.000539618
GM3 18:0/20:0	0.297392264	0.163702163	PE38:5	0.16539322	0.115390619
GM3 18:1/22:1	0.894676327	0.357870531	PE38:4	0.000153349	0.000707766
GM3 18:1/22:0	0.672669135	0.294599621	PE40p:6	7.74566E-06	0.00023237
GM3 18:0/22:0	0.069267533	0.069267533	PE40p:5	0.095127478	0.079272898
GM3 18:1/24:1	0.044349937	0.052176396	PE40p:4	0.084460612	0.07239481
GM3 18:1/24:0	0.18066045	0.117822033	PE40:6	0.000905914	0.003019715
GM3 18:0/24:0	0.821703282	0.335389095	PE40:5	0.018189498	0.026618777
PG32:1	0.799460988	0.335438177	PE40:6	0.015627866	0.024042871
PG34:2	0.109278352	0.085151963	PE42p:3	0.008893357	0.017786714
PG34:1	0.071564094	0.069255575	PE42p:2	0.263324875	0.153393131
PG36:4	0.783498133	0.331055549	PE42:9	0.636247174	0.284886794
PG36:3	0.068834853	0.070001545	PE42:8	0.689404986	0.299741298
PG36:2	0.160580645	0.113351043	PE42:7	0.597145286	0.273501658
PG36:1	0.322503927	0.174326447	LPC16e:0	0.001677382	0.005032145
LysoPI 16:1	0.538782995	0.254543147	LPC16:1	0.042278287	0.050733945
LysoPI 16:0	0.007219254	0.016042786	LPC16:0	0.704705929	0.304189609
LysoPI 18:1	0.021852125	0.029136166	LPC18:2	0.260078631	0.15298743
LysoPI 18:0	0.039180884	0.047976592	LPC18:1	0.01875711	0.026795871
LPA16:1	0.037351166	0.046688958	LPC18:0	0.052255074	0.058061193
LPA16:0	0.071171933	0.07000518	LPC20:0	0.097713461	0.07922713
LPA18:1	0.056381241	0.061506808	PC34:2	0.326832514	0.175088847
LPA18:0	0.890004968	0.358391262	PC34:1	0.018001473	0.027002209
PA32:3	0.110585094	0.085065457	PC36:4p	0.007268237	0.015574793
PA32:2	0.112967554	0.084725665	PC36:3p	0.049336943	0.056927242
PA32:1	0.252076991	0.151246194	PC36:2p	0.07873529	0.071577536
PA32:0	0.270054142	0.154316653	PC36:1p	0.35073228	0.182990755
PA34:2	0.275790668	0.154648972	PC36:0p	0.371736658	0.192277582
PA34:1	0.014035283	0.023392138	PC36:5	0.105967328	0.083658417
PA36:2	0.326990996	0.173623538	PC36:4	0.014060562	0.022800912
PA36:1	0.289220058	0.16067781	PC36:3	6.10349E-05	0.000457762
PA38:4	0.435062454	0.21935922	PC36:2	0.456198453	0.224359895
PI 32:1	0.32047003	0.174801835	PC36:1	0.066839588	0.070357461
PI 34:3	0.072372594	0.067849307	PC38:4p	0.333702164	0.175632718
PI 34:2	0.008254285	0.017077831	PC38:3p	0.25549966	0.151781976
PI 34:1	0.622713397	0.283051544	PC38:2p	0.451574976	0.223921476
PI 36:4	0.577079426	0.268409036	PC38:6	0.087245006	0.073728174
PI 36:3	0.014073412	0.022221177	PC38:5	4.23586E-06	0.000254152
PI 36:2	0.019535786	0.027259236	PC38:4	0.050416778	0.057075598
PI 36:1	0.809177385	0.337157244	PC38:3	0.078516262	0.072476549
PI 38:5	0.211594807	0.133638825	PC40:4p	0.001081673	0.00341581
PI 38:4	0.079298805	0.071013855	PC40:3p	0.000180096	0.000771842
PI 38:3	0.818863003	0.336519043	PC40:2p	0.028057249	0.036596412
PI 40:6	0.067798606	0.070136489	PC40:1p	0.633208114	0.285657796
PI 40:5	0.529440637	0.252114589	PC40:7	0.388325288	0.197453536
PI 40:4	0.923069343	0.366782521	PC40:6	0.733830168	0.314498643
			PC40:5	0.000140811	0.000704057
